# Exploring Secondary Amine Carnosine Derivatives: Design, Synthesis, and Properties

**DOI:** 10.3390/molecules29215083

**Published:** 2024-10-28

**Authors:** Angelica Artasensi, Sarah Mazzotta, Ines Sanz, Licheng Lin, Giulio Vistoli, Laura Fumagalli, Luca Regazzoni

**Affiliations:** 1 Department of Pharmaceutical Sciences, Università degli Studi di Milano, Via L. Mangiagalli 25, 20133 Milan, Italy; 2 Department of Chemistry, Università degli Studi di Milano, Via Golgi 19, 20133 Milan, Italy

**Keywords:** carnosine derivatives, HNE binding, secondary amine, carnosinase resistance, reactive carbonyl species, oxidative stress, AGE, ALE

## Abstract

Carnosine is a naturally occurring dipeptide that has been advocated by some authors as an interesting scaffold for the development of potential therapeutic agents in view of the positive outcomes of its supplementation in animal models of human diseases. Its mode of action seems to depend on the quenching of toxic electrophiles, such as 4–hydroxynonenal (HNE). However, carnosine’s bioavailability in humans is lower than that in other mammals. The main reason for such an unfavorable pharmacokinetic profile is the activity of the enzyme human serum carnosinase (E.C. 3.4.13.20), which rapidly hydrolyzes carnosine upon absorption. Therefore, some studies have focused on the design of carnosinase-resistant derivatives that retain binding activity toward toxic electrophiles. Nevertheless, the structural modification of the *N*-terminus amino group of carnosine has rarely been considered, possibly because of its key role in the electrophile scavenging mechanism. This was proven, since some carnosine *N*-terminus modification generated inactive compounds, despite some derivatives retaining oral bioavailability and gaining resistance to carnosinase hydrolysis. Herein, we therefore report a study aimed at exploring whether the amino group of carnosine can be conveniently modified to develop carnosinase-resistant derivatives retaining the dipeptide activity toward toxic electrophiles.

## 1. Introduction

The oxidation of biomolecules such as lipids, amino acids, and carbohydrates generates reactive carbonyl species (RCS), which contain carbonyl groups and are highly reactive with nucleophilic groups in proteins and nucleic acids [1,2]. RCS are produced under aerobic conditions through sugar oxidation and the lipid peroxidation of polyunsaturated fatty acids (PUFAs), leading to the formation of advanced glycoxidation and lipoxidation products (AGEs and ALEs). While RCS at physiological levels play roles in the immune response and cellular signaling, elevated levels result in carbonyl stress, contributing to the development of metabolic syndrome, neurodegenerative disorders, and chronic diseases, like diabetes and rheumatoid arthritis [3,4,5]. RCS are more stable and have longer lifespans than reactive oxygen species (ROS), and are also found in pollutants, pharmaceuticals, cigarette smoke, and food additives [6,7,8,9]. RCS can be classified into three groups based on their structure: α,β-unsaturated aldehydes, including acrolein and 4-hydroxy-*trans*-2-nonenal (HNE); dialdehydes, like glyoxal and malondialdehyde; and keto aldehydes, for example, methylglyoxal [2].

Human detoxification of reactive carbonyl species (RCS) relies on natural defense mechanisms involving phase I and II enzymes, such as cytochromes P450, aldehyde and alcohol dehydrogenases, or glutathione S-transferase [10,11,12,13,14,15]. However, developing sequestering agents for RCS is a key challenge in medicinal chemistry due to their involvement in various diseases and potential as drug targets. Over the years, many compounds able to react with RCS have been described by different research groups [16]. The most studied detoxifying agents are aminoguanidine, pyridoxamine, hydralazine, and carnosine. The first three are highly reactive against RCS, but with limited clinical use due to the absence of selectivity and subsequent cross-reaction with physiological aldehydes as pyridoxal [17]. Carnosine is a naturally occurring dipeptide (i.e., *β*-alanine-l-histidine) that is of relevant interest because it is involved in a variety of biological functions. It has two enantiomers (i.e., l- and d-carnosine), but only l-carnosine is absorbed through the gastrointestinal tract. Several studies demonstrated the positive effects of such a peptide in metabolic disorders such as diabetes, cancer, and neurodegenerative diseases like Alzheimer’s disease [18,19,20,21]. Many of these effects are related to antioxidant, anti-inflammatory, and free-radical/carbonyl scavenger activities. In fact, carnosine is very reactive toward α,β-unsaturated carbonyls and shows sequestering properties toward both acrolein and HNE, which are among the strongest electrophiles [22,23,24,25].

Carnosine activity depends on the reactivity of both its amino group and imidazole ring, since such moieties are involved in a multi-step reaction mechanism characterized by a Schiff base formation and a subsequent intramolecular Michael addition at the C3 of the α,β-unsaturated system, furnishing a covalent adduct [26,27]. Unfortunately, carnosine’s therapeutic uses are limited because of its low bioavailability, since such a peptide is quickly degraded by the enzyme serum carnosinase (E.C. 3.4.13.20), a specific dipeptidase that hydrolyzes carnosine in β-alanine and l-histidine [28,29,30].

For these reasons, many efforts have been devoted to designing and synthesizing new carnosine analogues with suitable activity against RCS and improved plasma stability, which might be useful for the development of new efficient detoxifying agents [31]. Carnosine derivatives reported in the literature have been obtained through different approaches: (i) the substitution of l-histidine with d-histidine [31,32,33]; (ii) the modification of the carboxylic function of the histidine by reduction [19,34], esterification [31,33], or conversion to substituted amides [35,36]; (iii) the modification of the amino group at the peptide *N*-terminus [37,38,39,40]; (iv) the introduction of side chains in β-alanine [41]; (v) the modification of the imidazole ring [42,43] or; (vi) a combination of some of these approaches. The possibility to modify the primary amine function has not been fully exploited, since it plays a crucial role for binding to active transporters responsible for carnosine absorption, besides being important also for carnosine activity [44,45,46]. For instance, *N*-acetilcarnosine, as well as di-methyl-balenine, are inactive against HNE, while other derivatives reported in the literature obtained from the conjugation of carnosine to salicylic acid or sugars were not tested for their binding activity toward RCS [40,47]. Other *N*-substituted carnosine derivatives were synthesized and tested for their binding activity toward HNE [37]. Secondary amines were inactive, whereas some derivatives with a hydrazide moiety in place of the *N*-terminus were more active than carnosine [48]. However, the stability of such compounds against the hydrolysis operated by human serum carnosinase has not been tested. The *N*-terminus conjugation was also used as a strategy to link carnosine to hyaluronic acid [49]. Such a product undergoes resistance to carnosinase hydrolysis and has some binding activity against acrolein in vitro. The molecule was also tested in several animal models with encouraging results, especially for reducing osteoarthritis and rheumatoid arthritis and inhibition of amyloid-β aggregation [49].

Interestingly, our group recently described the scavenging properties of the natural histidine-containing dipeptide l-Pro-l-His against HNE. Compared with l-carnosine, such a peptide demonstrated both greater activity and higher stability against carnosinase hydrolysis, since a half-life of about 30 min was measured in human serum [50].

Moreover, an example of a bioavailable *N*-substituted derivative of carnosine was already reported. Specifically, carnosine methyl ester having a protecting group at the *N*-terminus (i.e., Cbz-*N*-carnosine-O-methyl) was found in rat serum upon oral administration, mainly as a metabolite derived from the hydrolysis of methyl ester, while the protecting group at the *N*-terminus was pretty stable [31].

Taken together, these findings are encouraging as it appears that some modifications of the amino group of carnosine can generate compounds with good plasma stability without affecting absorption, while others ensured a retention of reactivity toward HNE.

In this work, we consequently designed and synthesized new *N*-substituted l-carnosine derivatives to further investigate the role of the *N*-terminus amino group and its nucleophilicity in the quenching mechanism of RCS. The aim was to obtain carnosine derivatives reactive toward HNE and stable against human serum carnosinase hydrolysis.

**Figure 1:**
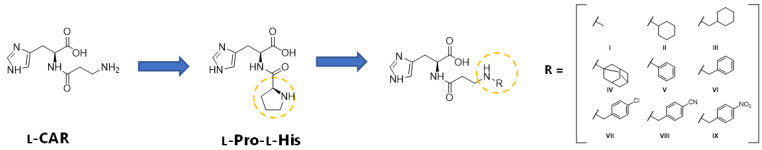
Rational design of the herein-reported carnosine derivatives (compounds: **I**–**IX**).

The synthesized compounds reported in Figure 1 have therefore been evaluated for their scavenging activity toward HNE and their metabolic stability in human serum. Additionally, the capability of interfering with carnosine hydrolysis was tested as well, as it is a key feature for the development of carnosinase inhibitors and few data are available on which modifications of carnosine’s structure are convenient for developing carnosinase inhibitors [44].

## 2. Results and Discussion

### 2.1. Chemistry

**Scheme 1:**
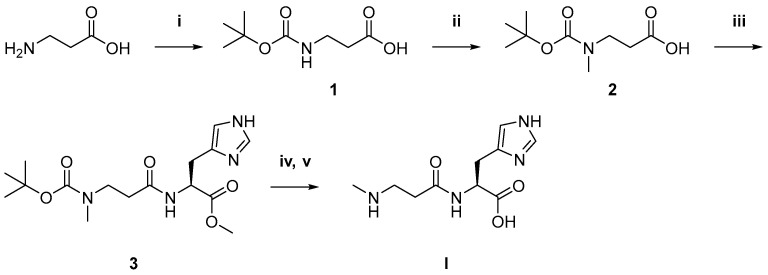
Synthetic pathway for the synthesis of compound **I**. Reagents and conditions: (i) di-*tert*-butyldicarbonate, NaOH, *t*-BuOH/H_2_O, rt, 24 h; (ii) iodomethane, NaH, THF, rt, 24 h; (iii) HBTU, DIPEA, l-histidine methyl ester, CH_3_CN, rt, 48 h; (iv) TFA, CH_2_Cl_2_, rt, 1 h; (v) LiOH, THF/H_2_O, rt, 4 h.

With the aim of investigating the impact of secondary amines and their nucleophilicity in binding reactive carbonyl species, variously *N*-substituted carnosine derivatives have been designed and synthesized, which differ in size, steric hindrance, and electronic properties of the substituent at the β-alanine amino group (Figure 1). -CH_3_, -Ph, -CH_2_Ph, -c-Hex, -CH_2_c-Hex -adamantyl, *p*-chloro-, *p*-ciano-, and *p*-nitrobenzyl groups have been inserted for this purpose. Three different synthetic routes have been employed for the generation of new compounds. The synthesis of the *N*-methyl derivative **I** started with the Boc protection of the β-alanine primary amine by the reaction with di-*tert*-butyldicarbonate under basic conditions, furnishing *N*-*tert*-butoxycarbonyl-3-aminopropanoic acid **1**. The next step involved the methylation reaction using iodomethane in presence of NaH, that provided *N*-*tert*-butoxycarbonyl-*N*-methyl-3-aminopropanoic acid (**2**). The coupling reaction between compound **2** and the amino acid l-histidine methyl ester in the presence of HBTU and DIPEA generated the intermediate *N*-*tert*-butoxycarbonyl-*N*-methyl-3-aminopropanoyl-l-histidinate **3**, which was doubly deprotected by the reaction with TFA and then with LiOH, afforded the desired compound *N*-methyl-3-aminopropanoyl-l-histidine **I** (Scheme 1).

**Scheme 2:**
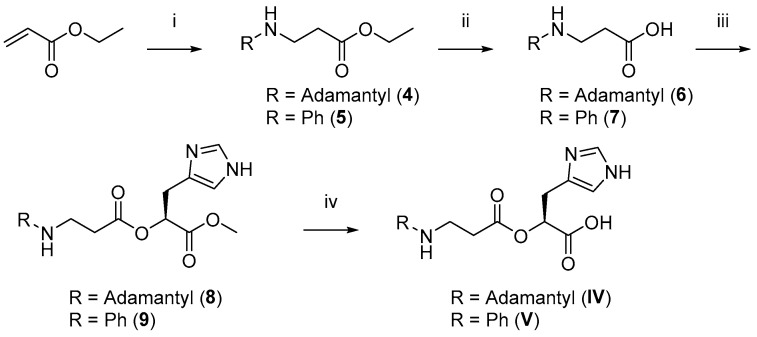
Synthetic pathway for the synthesis of compounds **IV** and **V**. Reagents and conditions: (i) adamantyl- (**4**) or phenylamine (**5**), AlCl_3_, 60 °C, 4 h; (ii) NaOH, MeOH, 65 °C, 4 h; (iii) HBTU, DIPEA, l-histidine methyl ester; CH_3_CN, rt, 48 h; (iv) LiOH, THF/H_2_O, rt, 4 h.

Compounds **IV** and **V** were prepared starting from commercially available ethyl acrylate. The first step via a Michael addition, using AlCl_3_ as a catalyst and the appropriate primary amine afforded ethyl *N*-(adamantan-1-yl)-3-aminopropanoate (**4**) and ethyl *N*-phenyl-3-aminopropanoate (**5**), which were subsequently hydrolyzed under basic conditions. Afterward, compounds **6** and **7** were coupled with l-histidine methyl ester under the same conditions as those described above, furnishing compounds **8** and **9**, respectively. The final compounds **V** (*N*-phenyl-3-aminopropanoyl-l-histidine) and **IV** (*N*-adamantyl-3-aminopropanoyl-l-histidine) were obtained by the hydrolysis of the methyl ester under basic conditions (Scheme 2).

**Scheme 3:**
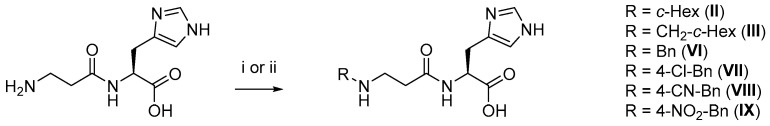
Synthetic pathway for the synthesis of compounds **II, III,** and **VI**–**IX**. Reagents and conditions: (i) cyclohexanone (**II**), NaCNBH_3_, EtOH, rt, 24 h. (ii) 2-cyclohexylacetaldehyde (**III**) or benzaldehyde (**VI**) or *p*-chlorobenzaldehyde (**VII**) or *p*-cyanobenzaldehyde (**VIII**) or *p*-nitrobenzaldehyde (**IX**), triethylamine, NaBH_4_, MeOH, rt, 24 h.

Finally, the synthesis of the other compounds (**II**, **III**, and **VI**–**IX**) was performed in one step through a reductive amination reaction of l-carnosine and the corresponding carbonyl compound in the presence of the appropriate reducing agent (Scheme 3). All new compounds were characterized by ^1^H NMR, ^13^C NMR, and MS analyses (see Section 3 and Supplementary Material).

### 2.2. Characterization of the Compounds

The compounds were first tested for their reactivity toward HNE according to an HPLC test previously used for the characterization of carnosine reactivity (see Section 3.2.1).

**Figure 2:**
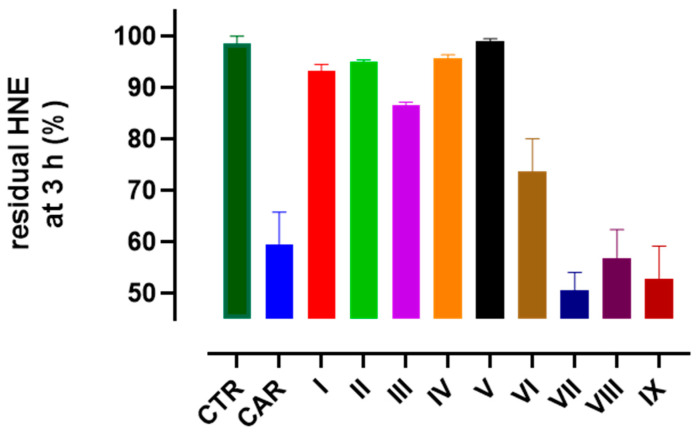
Residual concentration of HNE (50 µM in phosphate buffer, pH 7.4) after incubation for 3 h alone (CTR) and with a 20-fold excess of carnosine (CAR) or any of compounds **I**–**IX**.

As reported in Figure 2, compound **VI** was the most reactive molecule among the first series of carnosine derivatives synthesized (i.e., compounds **I**–**VI**, Figure 1). Specifically, the concentration of a 50 µM solution of HNE in phosphate buffer (pH 7.4) did not significantly decrease over time when incubated with compounds **I**–**V**; therefore, such molecules can be considered poorly or not reactive.

On the contrary, the residual amount of HNE decreased down to 73.7 ± 6.4 percent of the initial amount after incubation for 3 h with compound **VI**.

As reported for carnosine and other histidine-containing peptides, mass spectrometry was used to characterize the mechanism of reaction [27,50]. A signal at 473.27505 *m*/*z* that was increasing in intensity over time was detectable in high-resolution mass spectra from reaction batches containing compound **VI** and HNE. Such a signal is compatible with the monoprotonated ion of the Michael adduct between HNE and compound **VI** (473.27585 *m*/*z* theoretical value), similar to what has been observed for the reaction between HNE and carnosine or other histidine-containing peptides [27,50].

However, incubation with carnosine produced a greater decrease in HNE concentration over time, compared with compound **VI**. This suggests that compound **VI** is less active than carnosine, which was further confirmed by measuring kinetic parameters.

**Figure 3:**
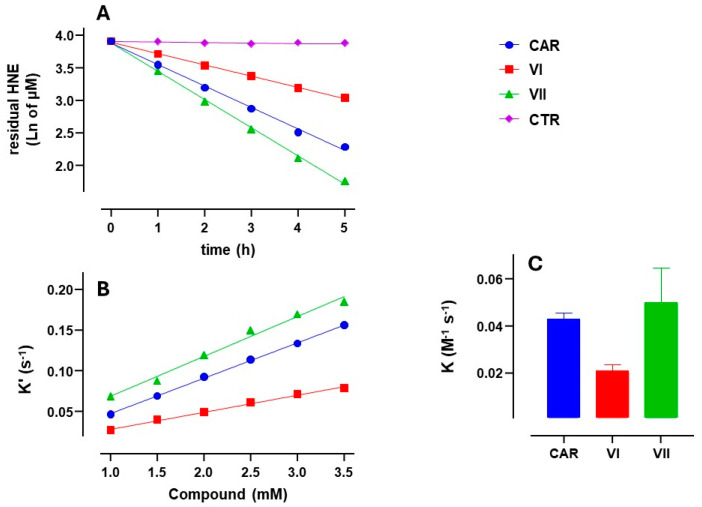
Kinetics data obtained for carnosine (CAR), compound **VI**, and compound **VII**. (**A**) Semilog plot of residual HNE (0.05 mM initial concentration) when incubated alone or with a 40-fold molar excess of compounds; (**B**) pseudo-first-order rate constants as a function of the concentration of compounds and (**C**) bar graph reporting the reaction rate constants.

Specifically, the reactions of HNE with carnosine and compound **VI** were found to proceed via second-order kinetics, with partial first-order kinetics in HNE concentration (i.e., coefficient of Equation (2), n = 1, see Section 3.2.2). This was confirmed since the concentration of HNE decreased linearly over time when pseudo-first-order kinetics were plotted using a semilog plot (see Figure 3A). The kinetics also have partial-first-order characteristics in carnosine concentration and in compound VI concentration (i.e., coefficient of Equation (2), m = 1, see Section 3.2.2), as the pseudo-first-order reaction rate constants increased linearly with the concentrations of carnosine and compound **VI** (see Figure 3B and Equation (3), Section 3.2.2).

The rate constant of the reaction between carnosine and HNE was estimated to be 0.043 (M^−1^ s^−1^, ±0.001 standard error), while the rate constant of the reaction between HNE and compound **VI** was estimated to be 0.021 (M^−1^ s^−1^, ±0.001 standard error, see Figure 3C). Welch’s ANOVA with Dunnett’s T3 multiple-comparison test rejected with 95% confidence the null hypothesis that carnosine and compound **VI** reacted with HNE according to the same reaction rate constant, which confirms that compound **VI** is less reactive than carnosine.

Despite the reduced reactivity, stability tests were performed on compound **VI**. Specifically, the molecule was spiked in human serum and its concentration was monitored over time to assess whether the compound was resistant to human serum carnosinase hydrolysis. In fact, the feature that most affects carnosine’s druggability is not its activity, but its instability in human serum [46].

**Figure 4:**
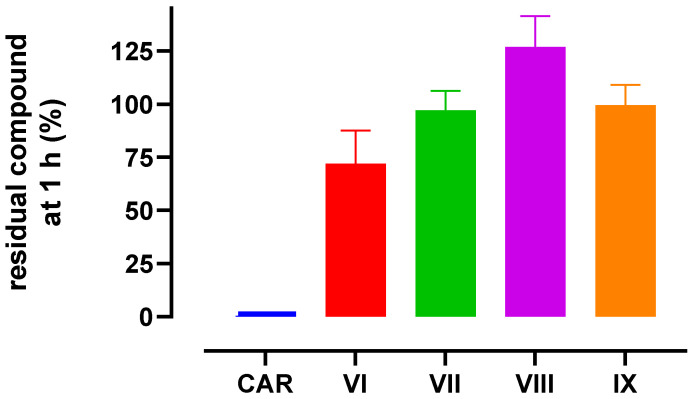
Residual concentrations of carnosine (CAR) and compounds **VI**–**IX**. Data were collected after incubation for 1 h in human serum at an initial concentration of 5 µM.

Compound **VI** was more stable than carnosine against carnosinase degradation, since 71.9 ± 15.6 percent of its initial amount was detectable in human serum after one hour, whereas no trace of carnosine could be found under the same experimental conditions (see Figure 4).

This is not surprising, since it has already been reported that any modification of the carboxylic or amino groups typically undergoes carnosinase resistance [19,31,34,51,52].

Since the stability against human carnosinase is an appealing feature for the design of bioactive carnosine derivatives [46], compounds **VII**–**IX** were designed to improve the HNE binding activity of compound **VI**, through the introduction of electron-withdrawing groups to modulate the nucleophilicity of secondary amine.

As predicted, all three compounds **VII**–**IX** were more active than compound **VI**. As reported in Figure 2, such compounds induced a greater reduction in the HNE concentration over time, with no significant difference between them. Moreover, all the compounds were stable against carnosinase hydrolysis (see Figure 4).

The activity of such compounds resembles carnosine kinetics, since the residual concentrations of HNE at different time points were similar for reaction batches containing carnosine or one of the compounds **VII**–**IX**. As reported in Figure 3A, the residual HNE was constantly lower for pseudo-first-order kinetics of compound **VII**. Compounds **VIII**–**IX** have superimposable kinetics. The rate constant of the reaction between compound **VII** and HNE was estimated to be 0.050 (M^−1^ s^−1^, ±0.006 standard error, see Figure 3C). However, Welch’s ANOVA with Dunnett’s T3 multiple comparison test accepted with 95% confidence the null hypothesis that carnosine and compound **VII** had the same reaction rate constant. This indicates that carnosine can be modified at the *N*-terminus to obtain compounds with unaltered or slightly higher activity toward HNE.

In order to validate the experimental data, theoretical studies on the nucleophilicity of l-carnosine and the most active secondary amines (**VI**–**IX**) were performed at the DFT/B3LYP/6-311g(d,p) level of theory. In addition, due to the unexpected poor reactivity of compound **I**, its nucleophilicity was also computed. The reactivity descriptor used for quantitative assessment of nucleophilicity at the nitrogen of the secondary amine is the relative nucleophilicity index (*f*_k_^−^). Fukui functions for electrophilic attack on an atom, *k*, in an *N*-electron system were introduced by Yang and Mortier [53] as:

*f*_k_^−^ = *q_k_*(N) − *q_k_*(N − 1) (1)

where *q_k_*(N) and *q_k_*(N − 1) are the atomic charges of the system with N and N − 1 electrons, respectively.

**Figure 5:**
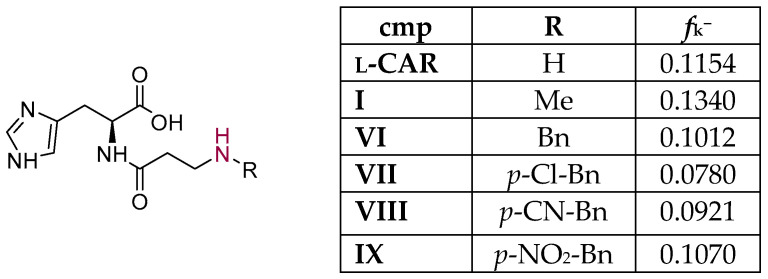
Quantitative assessment of nucleophilicity (*f*_k_^−^) at the nitrogen (colored in red) of the secondary amine for selected compounds.

The results are displayed in Figure 5:

The effect of the substituent on the nucleophilicity of l-carnosine is exhibited in the following order:

Me > H > Bn > *p*-NO_2_-Bn > *p*-CN-Bn > *p*-Cl-Bn

From the trend provided by the theoretical nucleophilicity scale, it can be easily concluded that the *f*_k_^−^ value is consistent with the experimental data by applying HSAB (hard and soft acids and bases) theory. As a matter of fact, lower Fukui values suggest harder nucleophilicity, which aligns with the fact that hard nucleophiles (with lower *f*_k_^−^) would better react with hard electrophiles, like the carbonyl group of HNE. The DFT results might show that soft nucleophiles (higher *f*_k_^−^ values) are more reactive overall, but in the specific case of a hard electrophile, the hard nucleophiles (with lower *f*_k_^−^) are favored experimentally [54]. Since, in related congeners, nucleophilicity parallels basicity, the obtained results also emphasize that less basic derivatives are more potent RCS scavengers due to the greater abundance of the reactive neutral amino group.

These findings demonstrate that it is possible to modify the *N*-terminus of carnosine to obtain derivatives totally resistant to carnosinase hydrolysis with retained reactivity toward HNE. Although the reactivity of compounds **VIII**–**IX** is in line with that of prolyl-histidine, they are indeed a more promising scaffold for the development of bioactive compounds, since prolyl-histidine is not completely stable in human serum [50].

Compounds **I**–**IX** were also tested for their ability to inhibit carnosine degradation operated by carnosinase. As no alteration of serum hydrolytic rate toward carnosine was observed, the stability in serum of such compounds is likely to be a consequence of a reduced binding affinity to the enzyme. This also suggests that the modification of the amino group of carnosine is possibly not a good strategy for the design of inhibitors of human serum carnosinase. Such a hypothesis is consistent with data suggesting the importance of *N*-terminus amino group for substrate recognition [44,45,46].

## 3. Materials and Methods

### 3.1. Chemistry

#### 3.1.1. General

All reagents and solvents were purchased from Merck KGaA, Darmstadt, Germany; they were analytical grade and used without further purification. Reactions were monitored by thin-layer chromatography (TLC) performed on precoated plates (Supelco^®^) and an UV detector for visualization. Purifications were performed by flash chromatography using silica gel (particle size 40–63 μm, Merck) on Isolera^TM^ (Biotage, Uppsala, Sweden) apparatus. NMR spectra were recorded at 25 °C using a Varian 300 Mercury spectrometer at 300 MHz for ^1^H NMR, and at 75.43 MHz for ^13^C NMR. The reported chemical shifts (δ) are presented in parts per million (ppm) and the coupling constants (*J*) are in hertz (Hz). The chemical shift values (δ) are referenced to the residual non-deuterated components of the NMR solvents (CD_3_OD, CDCl_3_, or D_2_O). The spin multiplicities are reported as s (singlet), d (doublet), t (triplet), q (quadruplet), m (multiplet), or br s (broad singlet). COSY experiments were performed to assign the signals in the NMR spectra. Melting points were determined by a Buchi Melting Point B-540 apparatus. The purity of all the final compounds was confirmed to be ≥95% by HPLC and identity was confirmed by high-resolution MS. Such data were obtained by using the same instruments as those described in Section 3.2.

#### 3.1.2. Synthesis of *N*-Tert-Butoxycarbonyl-3-Aminopropanoic Acid (**1**)

β-Alanine (250 mg, 2.80 mmol) was dissolved in a mixture of *t*-BuOH/H_2_O (2:1, 6 mL) and NaOH (123.17 mg, 3.08 mmol) was added to the flask, together with di-*tert*-butyl dicarbonate (672.21 mg, 3.08 mmol). The mixture was stirred overnight at room temperature and then diluted with H_2_O (2 mL) and extracted with cyclohexane (2 × 5 mL). The aqueous phase was cooled to 5 °C, adjusted to pH 4–5 with citric acid and extracted with ethyl acetate (3 × 3 mL). The organic layers were dried over Na_2_SO_4_, filtered, and evaporated under reduced pressure. The residue obtained was recrystallized from ethyl acetate/hexanes as white solid and used without further purification for the next reaction (quant yield). *R*_f_ = 0.76 (dichloromethane-methanol 9:1). ^1^H NMR (300 MHz, CDCl_3_) δ 3.44–3.35 (m, 2H), 2.63–2.54 (m, 2H), and 1.45 (m, 9H).

#### 3.1.3. *N*-Tert-Butoxycarbonyl-*N*-Methyl-3-Aminopropanoic Acid (**2**)

NaH (187.12 mg, 7.8 mmol) was suspended in dry THF (2 mL) and cooled to 0 °C under nitrogen. Then, a solution of compound **1** (590 mg, 3.12 mmol) in dry THF (3 mL) was added. After 5 min, iodomethane (0.49 mL, 7.8 mmol) in dry THF (2 mL) was also added to the flask. The reaction mixture was stirred at room temperature overnight and then quenched with cold H_2_O (5 mL) and washed with Et_2_O (5 mL). The aqueous phase was acidified and extracted with ethyl acetate (4 × 2 mL). The solution was dried over Na_2_SO_4_ and filtered. The organic phases were evaporated under a vacuum to obtain compound **9** (66% yield). *R*_f_ = 0.53 (dichloromethane-methanol 15:1). ^1^H NMR (300 MHz, CDCl_3_) δ 3.30 (t, *J* = 6.4 Hz, 2H), 2.60 (s, 3H), 2.30 (t, *J* = 6.4 Hz, 2H), 1.10 (m, 9H).

#### 3.1.4. Synthesis of Ethyl *N*-(Adamantan-1-Yl)-3-Aminopropanoate (**4**)

Ethyl acrylate (0.18 mL, 1.65 mmol) and AlCl_3_ (4.00 mg, 0.03 mmol, 2%mol) were added to a solution of adamantyl amine (249.56 mg, 1.65 mmol) in ethanol (2 mL). The reaction mixture was stirred at 40 °C for 4 h. Then, 15 mL of ethyl acetate was added and a precipitate formed. The precipitate was filtered and washed with cold ethyl acetate. Finally, the organic solvent was removed under reduced pressure to produce compound **5** as colorless oil (quant yield). *R*_f_ = 0.30 (cyclohexane/ethyl acetate 1:1). ^1^H NMR (300 MHz, CDCl_3_) δ 4.17–4.06 (m, 2H), 2.88 (t, *J* = 6.7 Hz, 2H), 2.53 (t, *J* = 6.7 Hz, 2H), 2.11–2.00 (m, 3H), 1.70–1.53 (m, 13H), 1.25 (t, *J* = 7.2 Hz, 3H).

#### 3.1.5. Synthesis of Ethyl *N*-Phenyl- 3-Aminopropanoate (**5**)

Aniline (0.39 mL, 4.29 mmol) was added to a mixture of ethyl acrylate (0.70 mL, 6.44 mmol) and AlCl_3_ (17.33 mg, 0.13 mmol). The reaction mixture was stirred at 60 °C for 4 h. Then, 30 mL of ethyl acetate was added and a precipitate formed. The precipitate was filtered and washed with cold ethyl acetate. Finally, the organic solvent was removed under reduced pressure to produce compound **6** as brown oil (85% yield). *R*_f_ = 0.62 (cyclohexane/ethyl acetate 2:1). ^1^H NMR (300 MHz, CDCl_3_): δ 7.19 (t, *J* = 7.8 Hz, 2H), 6.73 (t, *J* = 7.3 Hz, 1H), 6.64 (d, *J* = 7.7 Hz, 2H), 4.16 (q, *J* = 7.1 Hz, 2H), 3.46 (t, *J* = 6.4 Hz, 2H), 2.62 (t, *J* = 6.4 Hz, 2H), 1.26 (t, *J* = 7.1 Hz, 3H).

#### 3.1.6. General Procedure for the Synthesis of *N*-Substituted-3-Aminopropanoic Acid

A 10% aqueous solution of NaOH (4.13 mmol) was added to a solution of ethyl *N*-substituted-3-aminopropanoate (**4** and **5**) (3.61 mmol) in methanol (15 mL). The reaction mixture was refluxed at 65 °C and stirred until TLC showed that all the starting material had reacted (4 h). Then, the organic solvents were removed under vacuum and the residue was purified using an ion exchange resin (IRA-400) to obtain desired compounds.

*N-(Adamantan-1-yl)-3-aminopropanoic acid* (**6**): The product was obtained as colorless oil (quant yield). *R*_f_ = 0.14 (cyclohexane/ethyl acetate 1:1). ^1^H NMR (300 MHz, D_2_O) δ 3.17 (t, *J* = 6.7 Hz, 2H), 2.63 (t, *J* = 6.6 Hz, 2H), 2.11–2.02 (m, 3H), 1.80–1.71 (m, 6H), 1.67–1.48 (m, 6H).
*N-Phenyl-3-aminopropanoic acid* (**7**): The product was obtained as brown oil (97% yield). *R*_f_ = 0.18 (cyclohexane/ethyl acetate 2:1). ^1^H NMR (300 MHz, D_2_O): δ 7.17 (t, *J* = 7.5 Hz, 2H), 6.75 (d, *J* = 7.7 Hz, 3H), 3.22 (d, *J* = 6.4 Hz, 2H), 3.20 (t, *J* = 6.4 Hz, 2H), 2.37 (t, *J* = 6.4 Hz, 2H).

#### 3.1.7. General Procedure for the Synthesis of *N*-Substituted-3-Aminopropanoyl-l-Histidine Methyl Esters

*N-substituted-3-aminopropanoic acid* (**2**, **6** and **7**) (0.80 mmol) was dissolved in acetonitrile (~10 mL) and cooled to 0 °C. Then, HBTU (0.84 mmol, 318.57 mg) and DIPEA (0.88 mmol, 0.15 mL) were added, and the mixture was stirred at room temperature for 1 h (mixture 1). In the meantime, l-histidine methyl ester (0.80 mmol, 193.68 mg) was suspended in acetonitrile (15 mL) and after adding DIPEA (1.68 mmol, 0.29 mL) the mixture was stirred at room temperature for 1 h (mixture 2). Afterwards, mixture 2 was added dropwise to mixture 1 and stirred for 48 h. The solvent was evaporated under vacuum and the residue was dissolved in dichloromethane or ethyl acetate and washed with a 10% aqueous solution of NaHCO_3_ (15 mL or 3 mL) and NH_4_Cl (15 mL or 3 mL). The combined organic phases were dried over Na_2_SO_4_ and evaporated to dryness to obtain compounds **3**, **8**, and **9**.
*N-Tert-butoxycarbonyl-N-methyl-3-aminopropanoyl-L-histidine methyl ester* (**3**): Compound **10** was obtained as colorless oil and used without further purification for the next reaction (89% yield). *R*_f_ = 0.43 (ethyl acetate–methanol 8:2 + 1% NH_3_). ^1^H NMR (300 MHz, CDCl_3_) δ 7.26 (s, 1H), 6.91 (s, 1H), 5.29 (s, 1H), 4.86–4.71 (m, 1H), 3.78–3.60 (m, 5H), 3.15 (m, 2H), 2.80 (s, 3H), 2.45 (t, *J* = 6.6 Hz, 2H), 1.53–1.35 (m, 9H).
*N-(Adamantan-1-yl)-3-aminopropanoyl-l-histidine methyl ester* (**8**): The product was obtained as yellow pale oil and used without further purification for the next reaction (45% yield). *R*_f_ = 0.35 (dichloromethane–methanol 7:3). ^1^H NMR (300 MHz, D_2_O) δ 7.85 (s, 1H), 7.10 (s, 1H), 4.39–4.27 (m, 1H), 3.07–2.72 (m, 7H), 2.44 (t, *J* = 6.7 Hz, 2H), 2.06–1.89 (m, 3H), 1.73–1.37 (m, 12H).
*N-Phenyl-3-aminopropanoyl-l-histidine methyl ester* (**9**): Compound **5** was further purified by flash column chromatography using ethyl acetate–methanol (8:2) as an eluent and was obtained as brown oil (64% yield). *R*_f_ = 0.44 (ethyl acetate-methanol 8:2). ^1^H NMR (300 MHz, CDCl_3_) δ 7.52 (s, 1H), 7.14 (t, *J* = 7.6 Hz, 2H), 6.82 (s, 1H), 6.68 (m, 3H), 4.87–4.70 (m, 1H), 3.76–3.60 (m, 3H), 3.50–3.33 (m, 1H), 3.23–3.13 (m, 3H), 2.55 (t, *J* = 6.4 Hz, 2H).

#### 3.1.8. Synthesis of *N*-Methyl-3-Aminopropanoyl-l-Histidine (**I**)

TFA (12 mmol, 0.92 mL) was added dropwise to a cooled solution of compound **3** (0.8 mmol, 285 mg) in CH_2_Cl_2_ (15 mL). The reaction mixture was stirred for 1 h at room temperature. The solvent was evaporated and the residue dissolved in a mixture of THF/H_2_O (10:1, 11 mL). The solution was cooled to 0 °C with an ice bath, then LiOH 2M (4 mmol, 2 mL) was added and the reaction was stirred for 4 h at room temperature. The solvents were evaporated to dryness and the crude product was further purified by reverse phase column chromatography (6 g Sfär C18D) using water–acetonitrile (90:10) as the eluent, obtaining the pure compound as white resin (91% yield). *R*_f_ = 0.41 (water–acetonitrile 90:10). ^1^H NMR (300 MHz, D_2_O) δ 8.30 (s, 1H), 7.07 (s, 1H), 4.34 (m, 1H), 3.19–3.02 (m, 3H), 2.95 (dd, *J* = 15.4 Hz, *J* = 8.3 Hz, 1H), 2.70–2.48 (m, 5H). ^13^C NMR (75 MHz, D_2_O) δ 176.5, 171.3, 133.3, 118.2, 114.3, 54.2, 44.8, 32.9, 30.8, 27.1. C_10_H_16_N_4_O_3_ [M + H]^+^ = 241.13 *m*/*z*.

#### 3.1.9. General Procedure for the Synthesis of 3-Substuituted Propanoyl-l-Histidine

3-substituted propanoyl-l-histidine methyl ester (**8** or **9**) was dissolved in a mixture of THF-H_2_O (2.5 mL–0.2 mL) and cooled to 0 °C with an ice bath; then, LiOH 2M (4 mmol) was added and the reaction was stirred for 4 h. Subsequently, the solvents were removed and the residue was further purified by reverse-phase column chromatography.

*N-(Adamantan-1-yl)-3-aminopropanoyl-l-histidine* (**IV**): The product was further purified by reverse-phase column chromatography (6 g Sfär C18D) using water–acetonitrile (gradient elution from 90:10 to 80:20 + 0.1% formic acid) as an eluent. The aqueous phase was concentrated by freeze-drying (lyophilization) and the pure compound was obtained as a yellow pale solid (70% yield). *R*_f_ = 0.11 (water–acetonitrile 80:20 + 0.1% formic acid). Mp 96 °C. ^1^H NMR (300 MHz, D_2_O) δ 8.46 (s, 1H), 7.13 (s, 1H), 4.42–4.30 (m, 1H), 3.23–3.03 (m, 3H), 2.97 (dd, *J* = 15.4, 8.3 Hz, 1H), 2.54 (t, *J* = 6.5 Hz, 2H), 2.11–2.01 (m, 3H), 1.82–1.68 (m, *J* = 21.3 Hz, 6H), 1.67–1.42 (m, 6H). ^13^C NMR (75 MHz, D_2_O) δ 176.3, 171.3, 133.1, 129.6, 116.6, 57.8, 54.0, 37.9, 35.4, 34.8, 31.2, 28.7, 27.0. C_19_H_28_N_4_O_3_ [M + H]^+^ = 361.35 *m*/*z*.
*N-Phenyl-3-aminopropanoyl-l-histidine* (**V**): The product was further purified by reverse-phase column chromatography (6 g Sfär C18D) using water–acetonitrile (80:20) as an eluent. The aqueous phase was concentrated by freeze-drying and the pure compound was obtained as brown resin (85% yield). *R*_f_ = 0.28 (H_2_O-CH_3_CN 80:20). ^1^H NMR (300 MHz, D_2_O) δ 8.04 (s, 1H), 7.12 (t, *J* = 7.8 Hz, 2H), 6.92 (s, 1H), 6.70 (t, *J* = 7.3 Hz, 1H), 6.62 (d, *J* = 7.8 Hz, 2H), 4.33–4.27 (m, 1H), 3.19 (t, *J* = 6.4, 2H), 3.01 (dd, *J* = 15.5 Hz, *J* = 8.5 Hz, 1H), 2.85 (dd, *J* = 15.3 Hz, *J* = 8.6 Hz, 1H), 2.40 2.32 (m, 2H). ^13^C NMR (75 MHz, D_2_O) δ 176.80, 173.99, 147.44, 133.72, 129.43, 118.98, 116.77, 114.60, 57.34, 40.29, 35.17, 27.70. C_15_H_18_N_4_O_3_ [M + H]^+^ = 303.14 *m*/*z*.

#### 3.1.10. Synthesis of *N*-Cyclohexyl-3-Aminopropanoyl-l-Histidine (**II**)

Cyclohexanone (15 mmol, 2.17 mL) and NaCNBH_3_ (4.44 mmol, 279.01 mg) were added to a solution of l-carnosine dihydrochloride (0.44 mmol, 130.05 mg) in ethanol (7.00 mL). The reaction mixture was stirred overnight at room temperature; then, the solvent was evaporated under reduced pressure. The residue was dissolved in a 10% aqueous solution of NaOH and stirred for 1 h; then, it was diluted in water (5 mL) and extracted with ethyl acetate (2 × 10 mL). The pH of the aqueous phase was adjusted to 7 with formic acid and the solvent was concentrated under vacuum. The crude product was further purified by reverse-phase column chromatography (6 g Sfär C18D) using water–acetonitrile (70:30 + 1% formic acid) to produce compound **II** as white solid (66% yield). Mp 101 °C. *R*_f_ = 0.45 (water–acetonitrile 60:40+ 1% formic acid). ^1^H NMR (300 MHz, D_2_O) δ 7.69 (s, 1H), 6.85 (s, 1H), 4.36–4.24 (m, 1H), 3.16–3.07 (m, 2H), 3.05–2.90 (m, 2H), 2.84 (dd, *J* = 15.1, 8.7 Hz, 1H), 2.61–2.46 (m, 2H), 1.96–1.80 (m, 2H), 1.80–1.58 (m, 2H), 1.57–1.45 (m, 1H), 1.29–1.08 (m, 5H). ^13^C NMR (75 MHz, D_2_O) δ 174.4, 172.9, 134.6, 131.2, 118.2, 58.5, 50.3, 43.0, 36.5, 33.3, 29.1, 25.9, 24.7. C_15_H_24_N_4_O_3_ [M + H]^+^ = 309.27 *m*/*z*.

#### 3.1.11. General Procedure for the Synthesis of *N*-Substituted-3-Aminopropanoyl-l-Histidine

Et_3_N (0.23 mL, 1.65 mmol) and the corresponding aldehydes (i.e, cyclohexanecarboxaldehyde (**III**), benzaldehyde (**VI**), *p*-chlorobenzaldehyde (**VII**), *p*-cyanobenzaldehyde (**VIII**), *p*-nitrobenzaldehyde (**IX**)) (1.10 mmol) were added to a suspension of l-carnosine dihydrochloride (125 mg, 0.55 mmol) in methanol (5 mL). The reaction was stirred at room temperature for 30 min. Afterward, NaBH_4_ (62.42 mg, 1.65 mmol) was added to the flask and left to react for 1 h. The solvent was removed under reduced pressure.

*N-Cyclohexylmethyl-3-aminopropanoyl-l*-histidine (**III**): The residue was dissolved in NaOH (0.1 M) and extracted with Et_2_O. The aqueous-phase pH was adjusted to 6 with 10% HCl aqueous solution and extracted with ethyl acetate. The solvents were evaporated to dryness. The product was further purified by reverse-phase column chromatography (6 g Sfär C18D) using water–acetonitrile (65:35 + 0.1% formic acid) as an eluent. The aqueous phase was concentrated by freeze-drying and the pure compound was obtained as white solid (80% yield). *R*_f_ = 0.32 (water–acetonitrile 65:35 + 0.1% formic acid). Mp 102 °C. ^1^H NMR (300 MHz, D_2_O) δ 7.73 (s, 1H), 6.86 (s, 1H), 4.35–4.25 (m, 1H), 3.11 (t, 2H), 3.01 (dd, *J* = 15.1 Hz, *J* = 8.4, 1H), 2.84 (dd, *J* = 15.3 Hz, *J* = 8.4, 1H), 2.73 (d, *J* = 6.67 Hz, 2H), 2.59–2.51 (m, 2H), 1.67–1.45 (m, 6H), 1.21–0.97 (m, 3H), 0.94–0.73 (m, 2H). ^13^C NMR (75 MHz, D_2_O) δ 176.3, 171.4, 133.1, 129.4, 116.6, 54.0, 53.4, 43.6, 34.3, 30.7, 29.7, 27.0, 25.4, 24.8. C_16_H_26_N_4_O_3_ [M + H]^+^ = 323.27 *m*/*z*.
*N-Benzyl-3-aminopropanoyl-l-histidine* (**VI**): The residue was dissolved in NaOH (0.1 M) and extracted with Et_2_O. The aqueous phase pH was adjusted to 6 with 10% HCl aqueous solution and extracted with ethyl acetate. The solvents were evaporated to dryness. The product was further purified by reverse phase column chromatography (6 g Sfär C18D) using water–acetonitrile (gradient elution from 90:10 to 70.30) as an eluent. The aqueous phase was concentrated by freeze-drying and the pure compound was obtained as white solid (55% yield). *R*_f_ = 0.26 (water–acetonitrile 90:10). Mp 103 °C. ^1^H NMR (300 MHz, D_2_O) δ 8.45 (s, 1H), 7.40–7.29 (m, 5H), 7.12 (s, 1H), 4.41–4.36 (m, 1H), 4.13 (s, 2H) 3.25–3.05 (m, 3H), 2.96 (dd, *J* = 15.1 Hz, *J* = 8.6 Hz, 1H), 2.66–2.50 (m, 2H). ^13^C NMR (75 MHz, D_2_O) δ 175.9, 171.3, 133.1, 130.4, 129.7, 129.6, 129.4, 129.2, 116.6, 53.8, 51.0, 42.6, 30.8, 26.9. C_16_H_20_N_4_O_3_ [M + H]^+^ = 317.16 *m*/*z*.
*N-(4-Chlorobenzyl)-3-aminopropanoyl-l-histidine* (**VII**): The residue was dissolved in Et_2_O (7 mL), the solid was removed by filtration, and the solvent was evaporated under reduced pressure to obtain the pure product as a white solid (53% yield). *R*_f_ = 0.30 (methanol–acetonitrile 8:2). Mp 97 °C. ^1^H NMR (300 MHz, CD_3_OD) δ 7.45 (s, *J* = 1.0 Hz, 1H), 7.30 (s, 4H), 6.80 (s, 1H), 4.48 (dt, *J* = 11.8, 5.9 Hz, 1H), 3.70 (s, 2H), 3.16 (dd, *J* = 15.0, 4.5 Hz, 1H), 2.98 (dd, *J* = 15.0, 7.6 Hz, 1H), 2.80 (t, *J* = 6.6 Hz, 2H), 2.40 (t, *J* = 6.6 Hz, 2H). ^13^C NMR (75 MHz, CD_3_OD) δ 176.94, 172.48, 137.99, 134.47, 132.45, 129.73, 128.09, 54.75, 51.97, 44.60, 35.12, 29.17. C_16_H_19_ClN_4_O_3_ [M + H]^+^ = 351.09 *m*/*z*.
*N-(4-Cyanobenzyl)-3-aminopropanoyl-l-histidine* (**VIII**): The residue was dissolved in Et_2_O (7 mL), the solid was removed by filtration, and the solvent was evaporated under reduced pressure to obtain the pure product as a white solid (55% yield). *R*_f_ = 0.27 (methanol–acetonitrile 8:2). Mp 101 °C. ^1^H NMR (300 MHz, CD_3_OD) δ 7.67 (d, J = 8.1 Hz, 2H), 7.51 (d, J = 8.1 Hz, 2H), 7.46 (s, 1H), 6.80 (s, 1H), 4.50 (dd, J = 7.3, 4.5 Hz, 1H), 3.81 (s, 2H), 3.16 (dd, J = 14.9, 4.6 Hz, 1H), 3.00 (dd, J = 14.9, 7.5 Hz, 1H), 2.80 (t, J = 6.5 Hz, 2H), 2.40 (t, J = 6.5 Hz, 2H). ^13^C NMR (75 MHz, CD_3_OD) δ 176.60, 172.43, 145.52, 134.29, 131.88, 128.90, 118.40, 110.27, 54.54, 52.25, 44.71, 35.23, 29.01. C_17_H_19_N_5_O_3_ [M + H]^+^ = 342.17 *m*/*z*.
*N-(4-Nitrobenzyl)-3-aminopropanoyl-l-histidine* (**IX**): The residue was dissolved in Et_2_O (7 mL), the solid was removed by filtration, and the solvent was evaporated under reduced pressure to obtain the pure product as a white solid (75% yield). *R*_f_ = 0.25 (methanol–acetonitrile 8:2). Mp 101 °C. ^1^H NMR (300 MHz, CD_3_OD) δ 8.17 (d, *J* = 8.4 Hz, 2H), 7.56 (d, *J* = 8.4 Hz, 2H), 7.47 (s, 1H), 6.81 (s, 1H), 4.50 (dd, *J* = 7.3, 4.7 Hz, 1H), 3.85 (s, 2H), 3.16 (dt, *J* = 12.0, 6.0 Hz, 1H), 3.06–2.93 (m, 1H), 2.82 (t, *J* = 6.3 Hz, 2H), 2.42 (t, *J* = 6.5 Hz, 2H). ^13^C NMR (75 MHz, CD_3_OD) δ 176.79, 172.51, 147.52, 147.02, 134.39, 128.90, 123.06, 54.66, 51.98, 44.75, 35.30, 29.17. C_16_H_19_N_5_O_5_ [M + H]^+^ = 362.09 *m*/*z*.

### 3.2. Analytical Methods

#### 3.2.1. HNE Binding Activity

The compounds were separately tested to determine their reactivity toward HNE. The assay was performed in triplicate by following an HPLC-UV method that had already been published. The stock solutions of the compounds were prepared in methanol (10 mM), since the compounds were very soluble in such a solvent. The compounds were then diluted down to the final concentration in phosphate buffer to follow the procedure reported in the literature [41,50]. Any reaction batch where a significant decrease in the HNE concentration over time was also analyzed by high-resolution mass spectrometry to verify the formation of a covalent adduct between the tested compound and HNE according to a procedure that had already published [50].

#### 3.2.2. Determination of the Reaction Rate Constants

The pseudo-first-order method [55], also known as the flooding method [56], was used to determine the kinetic parameters (i.e., reaction rate constant and reaction order) of the reaction between carnosine and HNE. Specifically, carnosine was the first histidine peptide reported to react with HNE [27]. The reaction is bimolecular, so the reaction rate is expected to depend on the concentration of both reagents, as in Equation (2).

(2) $$v=k\times {\left[carnosine\right]}^{m}\times {\left[HNE\right]}^{n}$$

When a molar excess of carnosine is used, the reaction rate depends solely on residual HNE (i.e., pseudo-first-order reaction, see Equation (4)):

(3) $$if\;\left[carnosine\right]\gg \left[HNE\right]\to \;k^{\prime }=k\times {\left[carnosine\right]}^{m}$$

(4) $$\to \;v=k^{\prime }\times {\left[HNE\right]}^{n}$$

Five separate reaction batches were prepared while maintaining a fixed concentration of HNE (50 µM), while carnosine was added at different concentrations, but keeping a molar excess to provide pseudo-first-order kinetics for all the reaction batches (1 to 3 mM). The HNE residual concentration was measured within 5 h by HPLC, as reported in Section 3.2.1. The HNE peak areas were transformed into concentration by using a calibration curve obtained from the analysis of standards prepared by the serial dilution of HNE down to concentrations ranging from 1 to 60 µM. Samples for kinetics and calibration curve were prepared in triplicate.

Data were plotted according to graphs expected to provide a linear trend for kinetics of order zero (i.e., [HNE] vs. time), one (i.e., Ln [HNE] vs. time), and two (i.e., 1/[HNE] vs. time). The partial order in HNE concentration (i.e., coefficients n of Equation (2)) was assigned by the graph that better fits a linear regression model and the slopes obtained for kinetics with different concentrations of HNE were used to calculate the pseudo-first-order reaction rate constants (i.e., κ′ of Equation (4)). Such constants were then plotted against the concentration of carnosine used for different experiments and data fitted to determine the reaction rate constant (i.e., κ, Equation (3)) and the partial order in carnosine concentration (i.e., coefficient m of Equation (2)). The same approach was repeated to obtain kinetics parameters of any compound with an appealing HNE binding ability, as in the assay reported in Section 3.2.1.

#### 3.2.3. Serum Stability

Serum stability was measured by spiking the compounds separately in pre-warmed (37 °C) serum aliquots down to a final concentration of 5 µM, as previously reported [50]. Minor modifications were applied to the method. Specifically, hydrolysis was blocked by the addition of trichloroacetic acid down to 2.5% to induce protein precipitation. The residual concentration of the compounds was then measured in the supernatant obtained upon centrifugation. Separate samples were prepared to determine the residual concentration at different time points. The supernatants were then analyzed by using a Surveyor LC system coupled with a Orbitrap ELITE mass spectrometer through an HESI electrospray interface (Thermo Fisher Scientific, Rodano, MI, Italy). Analyte separation was performed by using a Kinetex C18 column (150 mm × 2.10 mm, particle size 2.6 µm, pore size 100 Å, Phenomenex, Castel Maggiore, Italy). Elution was provided by using water and acetonitrile at 200 µL/min. The acetonitrile concentration was increased from 5 to 50 in 9 min, followed by a six-minute program to re-equilibrate the column to the initial condition before the next analysis. A solution of aqueous ammonium formate/formic acid was mixed with the mobile phase to ensure a constant ionic strength of 10 mM ammonium formate and to adjust the pH to 4.

During the elution, the nebulization and ionization was provided by the electrospray interface with the application of 3.5 kV, 30 units of sheath and auxiliary gas, 200 °C auxiliary gas temperature, and 300 °C capillary temperature. The analyzer was operated at a resolution of 30,000 (FWHM at 400 *m*/*z*) by using default settings and a scan range between 150 and 700 *m*/*z*. Peak areas were extracted as a single-ion chromatogram in a *m*/*z* window within 10 ppm from the theoretical monoprotonated ion of each compound.

The residual concentration of the compounds was estimated as the ratio of peak areas using the peak area of the measured sample as the antecedent term of the ratio and the peak area of a reference sample as the consequent term of the ratio. Reference samples were prepared by deproteinization of serum immediately after the beginning of the incubation period.

#### 3.2.4. Inhibition of Serum Carnosinase

The inhibition of serum carnosinase was measured following a previously reported method [57] with minor modifications. In detail, sample preparation was as reported in the literature, except for protein precipitation, which was performed by the addition of trichloroacetic acid down to a 2.5% final concentration. The supernatants were neutralized with phosphate buffer and the residual carnosine was derivatized with ortho-phtalaldehyde (OPA) to produce a stable phalimidine adduct [58]. Quantification of residual carnosine was performed by determining the carnosine–OPA adduct with the same method used to determine serum stability of carnosine derivatives (see Section 3.2.3).

### 3.3. Computational Details

The reactivity of the secondary amines was examined through DFT calculations using the B3LYP function combined with the 6-311g(d,p) standard basis set [59]. All data were analyzed with the Gaussian 16 program [60] and the graphical interface GaussView Version 6.1 [61] was used for the visualization. Since the experimental reactions to evaluate the impact of secondary amines in quenching reactive carbonyl species occurred in human serum, the water effect was taken into account by using self-consistent reaction field (SCRF). The Fukui functions were calculated in order to gain information associated with the local reactivity properties of the title compounds via Multiwfn 3.8 tool [62,63].

### 3.4. Data Analysis and Statistics

Data elaboration, analysis, and statistics were performed by using Microsoft Excel 2010 and Prism (v9.0, GraphPad Software LLC, https://www.graphpad.com, accessed on 24 October 2024).

## 4. Conclusions

Carnosine modification is considered one strategy to develop bioactive compounds mimicking, in humans, the beneficial effects observed upon carnosine supplementation in animal models of human disease [18,21,46]. Encouraging results have been already obtained through the modification of the carboxylic moiety of carnosine. Specifically, carnosinol was successfully tested both in vivo and in vitro [19,34]. Despite some model of structure–activity relationships describing the amino group as a moiety that is required for ensuring carnosine absorption [44,45,46], an example of a bioavailable *N*-substituted derivative of carnosine has already been reported [31]. Moreover, the absorption of active carnosine derivatives nowadays is less impacting on design and development, since there are several strategies to enhance the bioavailability [64]. Therefore, the major concern around the modification of *N*-terminus of carnosine remains the potential loss of activity that was observed in some studies [27,46]. In this context, the data reported herein prove that carnosine amino group can be conveniently modified to obtain derivatives with a retained activity toward HNE and improved stability against carnosinase hydrolysis. Among all carnosine derivatives tested so far, carnosinol is the only carnosine derivative showing both stability against carnosinase hydrolysis and enhanced activity against HNE [16,19,34]. Although the molecules herein described are apparently just as active as carnosine, this finding is encouraging, since some beneficial effects have been reported in humans after the administration of histidine peptides like anserine [65,66,67,68], which is less active than carnosine against HNE, but slightly more stable against carnosinase hydrolysis [27,57]. Therefore, the design of secondary amino derivatives of carnosine appears to be an alternative and appealing strategy for the development of bioactive compounds from the carnosine scaffold.

## Data Availability

Dataset available on request from the authors.

## Supplementary Materials

The following supporting information can be downloaded at: https://www.mdpi.com/article/10.3390/molecules29215083/s1, Figure S1: ^1^H and ^13^C NMR spectra of compound **I** (D_2_O); Figure S2: ^1^H and ^13^C NMR spectra of compound **II** (D_2_O); Figure S3: ^1^H and ^13^C NMR spectra of compound **III** (D_2_O); Figure S4: ^1^H and ^13^C NMR spectra of compound **IV** (D_2_O); Figure S5: ^1^H and ^13^C NMR spectra of compound **V** (D_2_O); Figure S6: ^1^H and ^13^C NMR spectra of compound **VI** (D_2_O); Figure S7: ^1^H and ^13^C NMR spectra of compound **VII** (D_2_O); Figure S8: ^1^H and ^13^C NMR spectra of compound **VIII** (D_2_O); Figure S9: ^1^H and ^13^C NMR spectra of compound **IX** (D_2_O); Table S1: Hirshfeld charges, condensed Fukui functions and condensed dual descriptors for **L-CAR**; Table S2: Hirshfeld charges, condensed Fukui functions and condensed dual descriptors for compound **I**; Table S3: Hirshfeld charges, condensed Fukui functions and condensed dual descriptors for compound **VI**; Table S4: Hirshfeld charges, condensed Fukui functions and condensed dual descriptors for compound **VII**; Table S5: Hirshfeld charges, condensed Fukui functions and condensed dual descriptors for compound **VIII**; Table S6: Hirshfeld charges, condensed Fukui functions and condensed dual descriptors for compound **IX**.

## Abbreviations

*tert*-butanol (*t*-BuOH); tetrahydrofuran (THF); trifluoroacetic acid (TFA); diisopropylamine (DIPEA); diethylether (Et_2_O); hexafluorophosphate benzotriazole tetramethyl uronium (HBTU); triethylamine (Et_3_N).
