# Supplementary material for: Exploring Secondary Amine Carnosine Derivatives: Design, Synthesis, and Properties
Source: Molecules. 2024 Oct 28;29(21):5083. doi: 10.3390/molecules29215083 (PMC11547551; doi:10.3390/molecules29215083)
Supplement: Supplementary file 1 [file molecules-29-05083-s001.zip › molecules-3259791-supplementary.pdf]

Supporting Information

Exploring secondary amines carnosine derivatives: design, synthesis and properties

Angelica Artasensi[1,§], Sarah Mazzotta[1,2,§], Ines Sanz[1], Giulio Vistoli [1], Laura Fumagalli[1*], Luca Regazzoni[1*]

§ share first authorship

[1]Department of Pharmaceutical Sciences, Università degli Studi di Milano, Via L. Mangiagalli 25, 20133 Milan, Italy

[2]Department of Chemistry, Università degli Studi di Milano, Via Golgi 19, 20133 Milan, Italy

* Correspondence: L.F., laura.fumagalli@unimi.it ; L.R., luca.regazzoni@unimi.it
*Laura Fumagalli is the corresponding author for the chemistry part and Luca Regazzoni is the corresponding author for the analytical part.

		Page No.
**Figure S1.**	^{1}H- and ^{13}C NMR spectra of compound **I**	S2
**Figure S2.**	^{1}H- and ^{13}C NMR spectra of compound **II**	S3
**Figure S3.**	^{1}H- and ^{13}C NMR spectra of compound **III**	S4
**Figure S4.**	^{1}H- and ^{13}C NMR spectra of compound **IV**	S5
**Figure S5.**	^{1}H- and ^{13}C NMR spectra of compound **V**	S6
**Figure S6.**	^{1}H- and ^{13}C NMR spectra of compound **VI**	S7
**Figure S7**	^{1}H- and ^{13}C NMR spectra of compound **VII**	S8
**Figure S8**	^{1}H- and ^{13}C NMR spectra of compound **VIII**	S9
**Figure S9**	^{1}H- and ^{13}C NMR spectra of compound **IX**	S10
**Table S1**	Hirshfeld charges, condensed Fukui functions and condensed dual descriptors for **L-CAR**	S11
**Table S2**	Hirshfeld charges, condensed Fukui functions and condensed dual descriptors for compound **I**	S12
**Table S3**	Hirshfeld charges, condensed Fukui functions and condensed dual descriptors for compound **VI**	S13
**Table S4**	Hirshfeld charges, condensed Fukui functions and condensed dual descriptors for compound **VII**	S15
**Table S5**	Hirshfeld charges, condensed Fukui functions and condensed dual descriptors for compound **VIII**	S17
**Table S6**	Hirshfeld charges, condensed Fukui functions and condensed dual descriptors for compound **IX**	S19

NMR spectra have been recorded at 300 MHz for ^{1}H-NMR, and at 75.43 MHz for ^{13}C-NMR.

Figure S1. ^{1}H- and ^{13}C NMR spectra of compound **I** (D_2O)

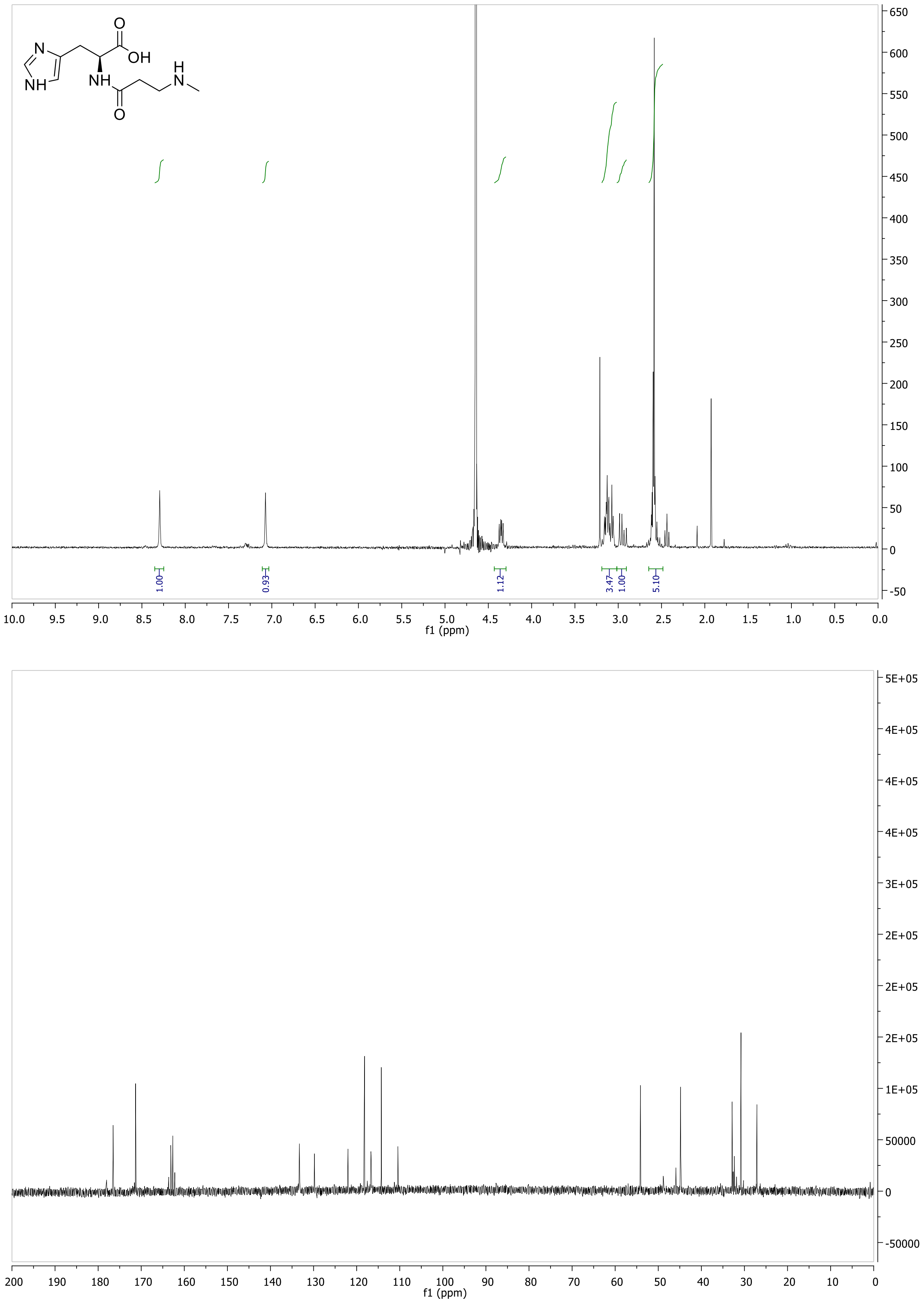

Figure S2. ^{1}H- and ^{13}C NMR spectra of compound **II** (D_2O)

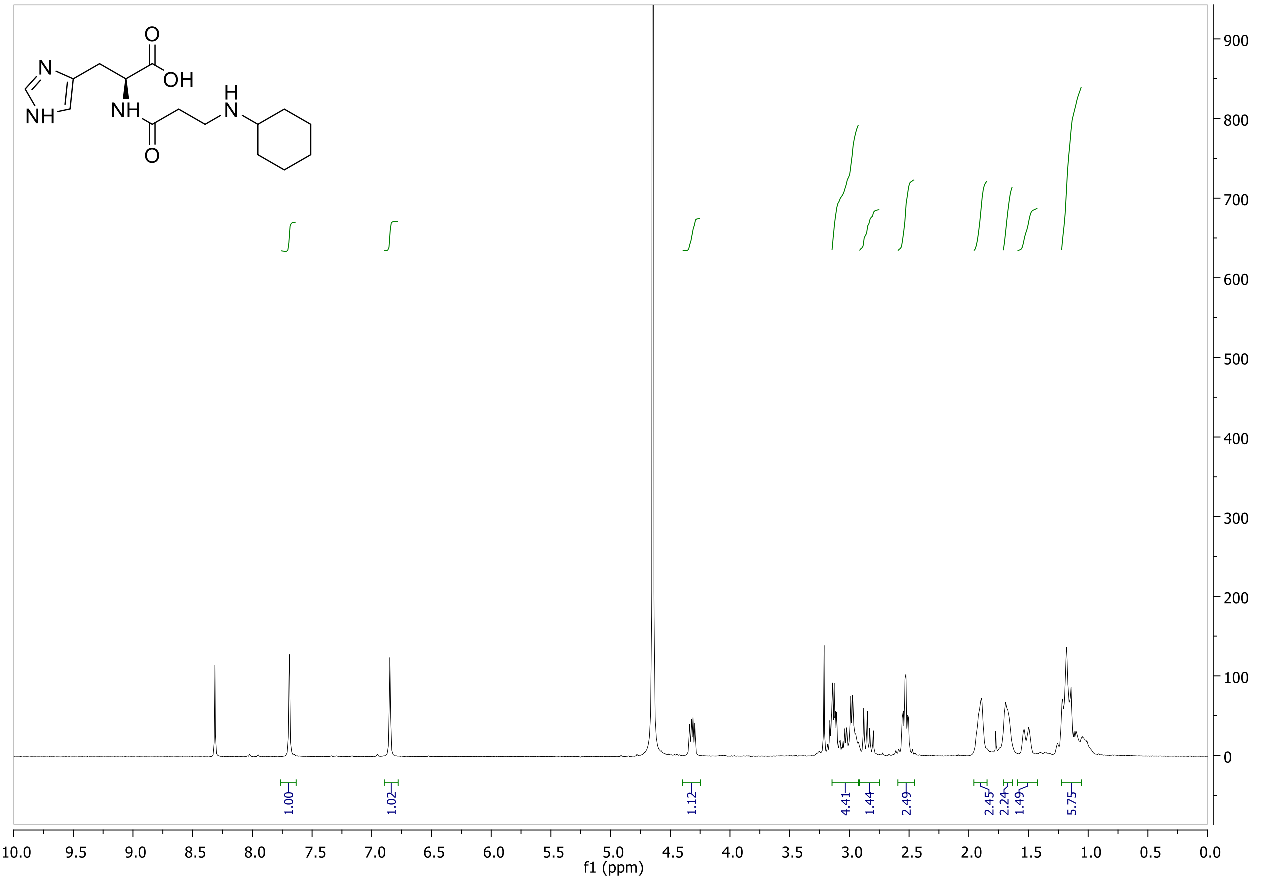

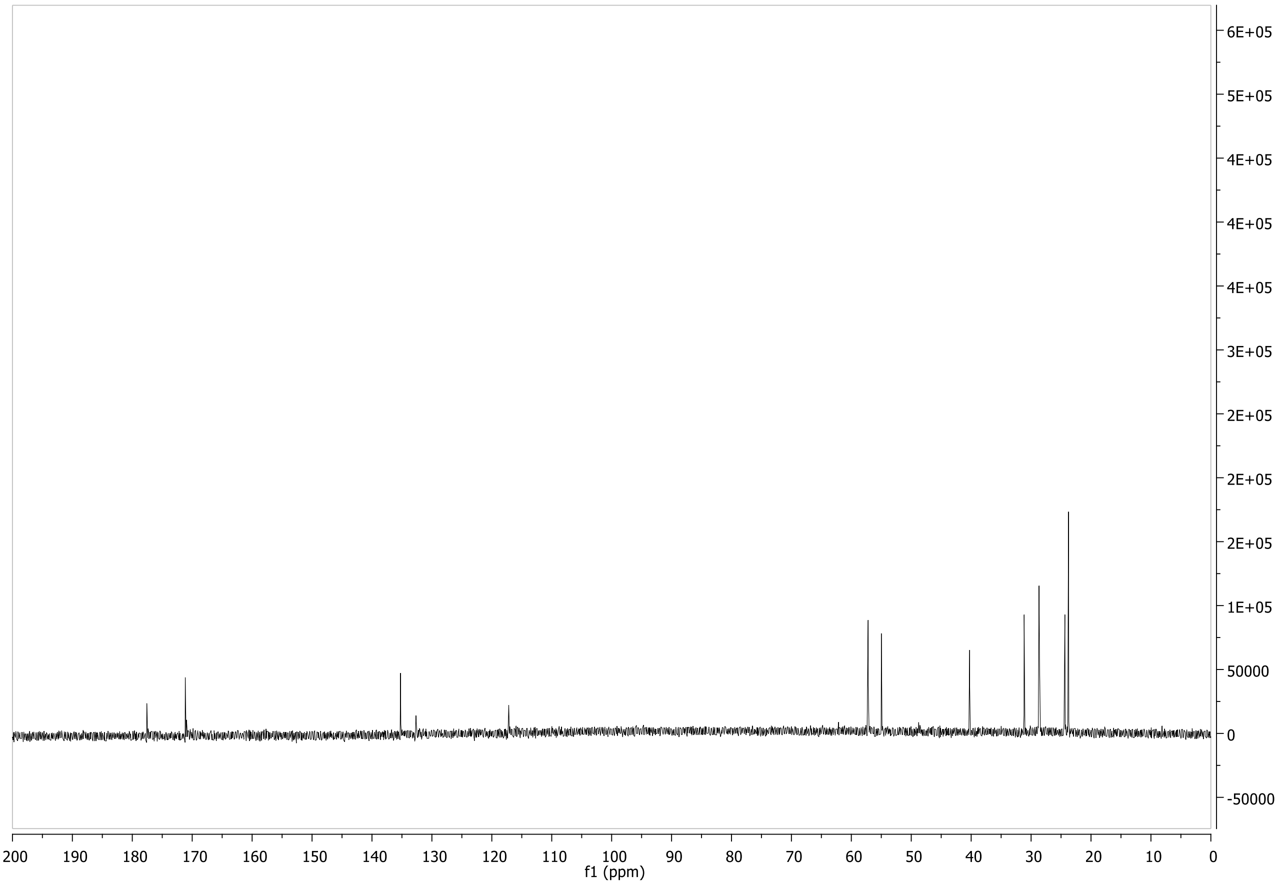

Figure S3. ^{1}H- and ^{13}C NMR spectra of compound **III** (D_2O)

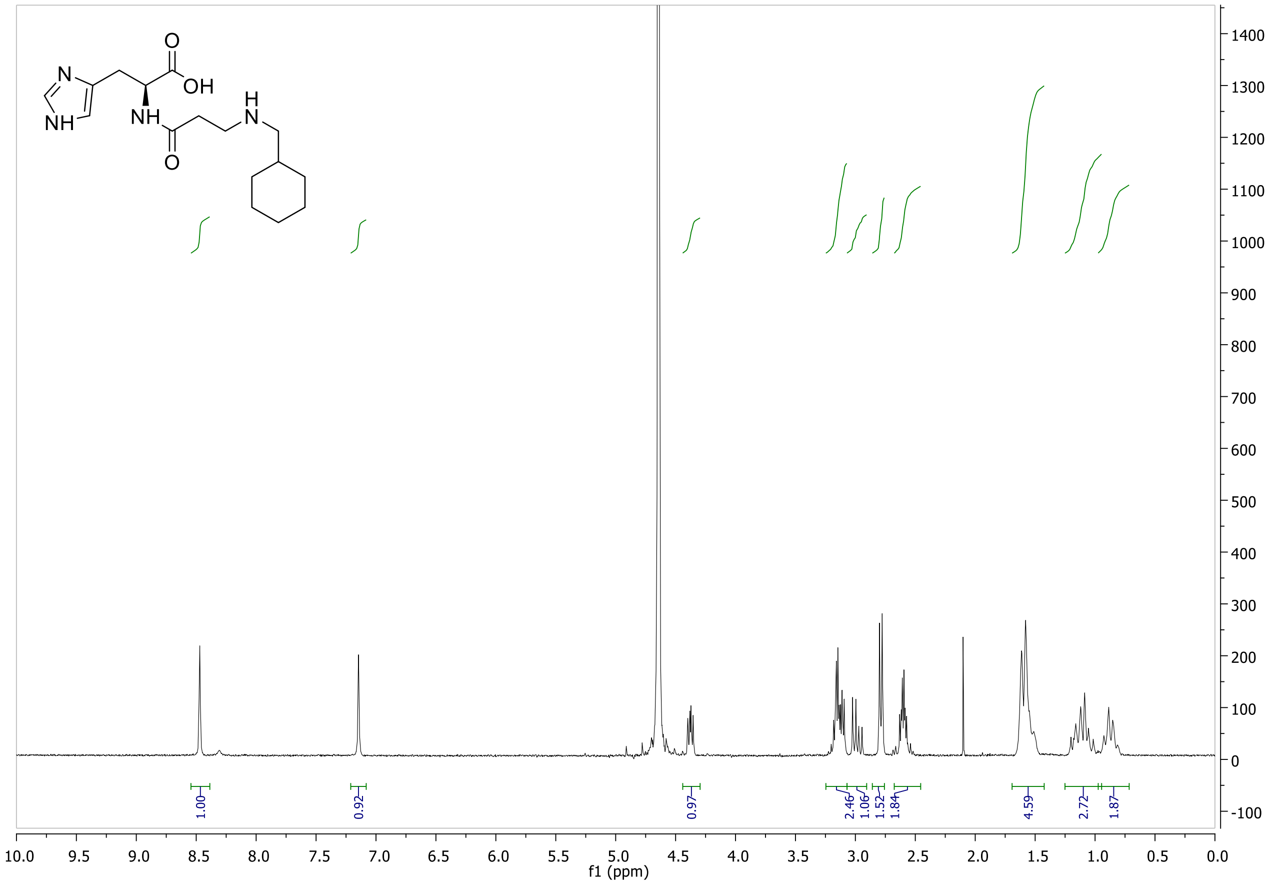

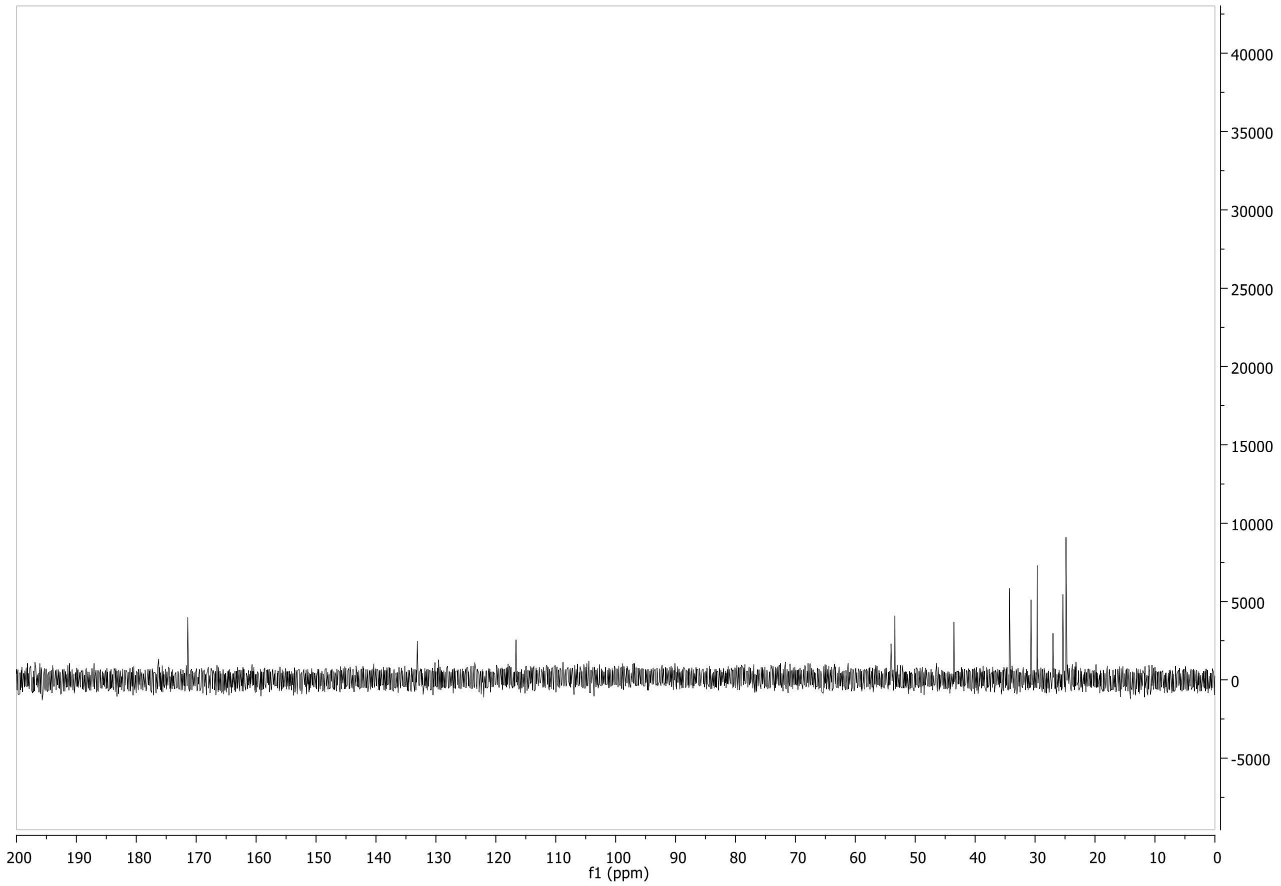

Figure S4. ^{1}H- and ^{13}C NMR spectra of compound **IV** (D_2O)

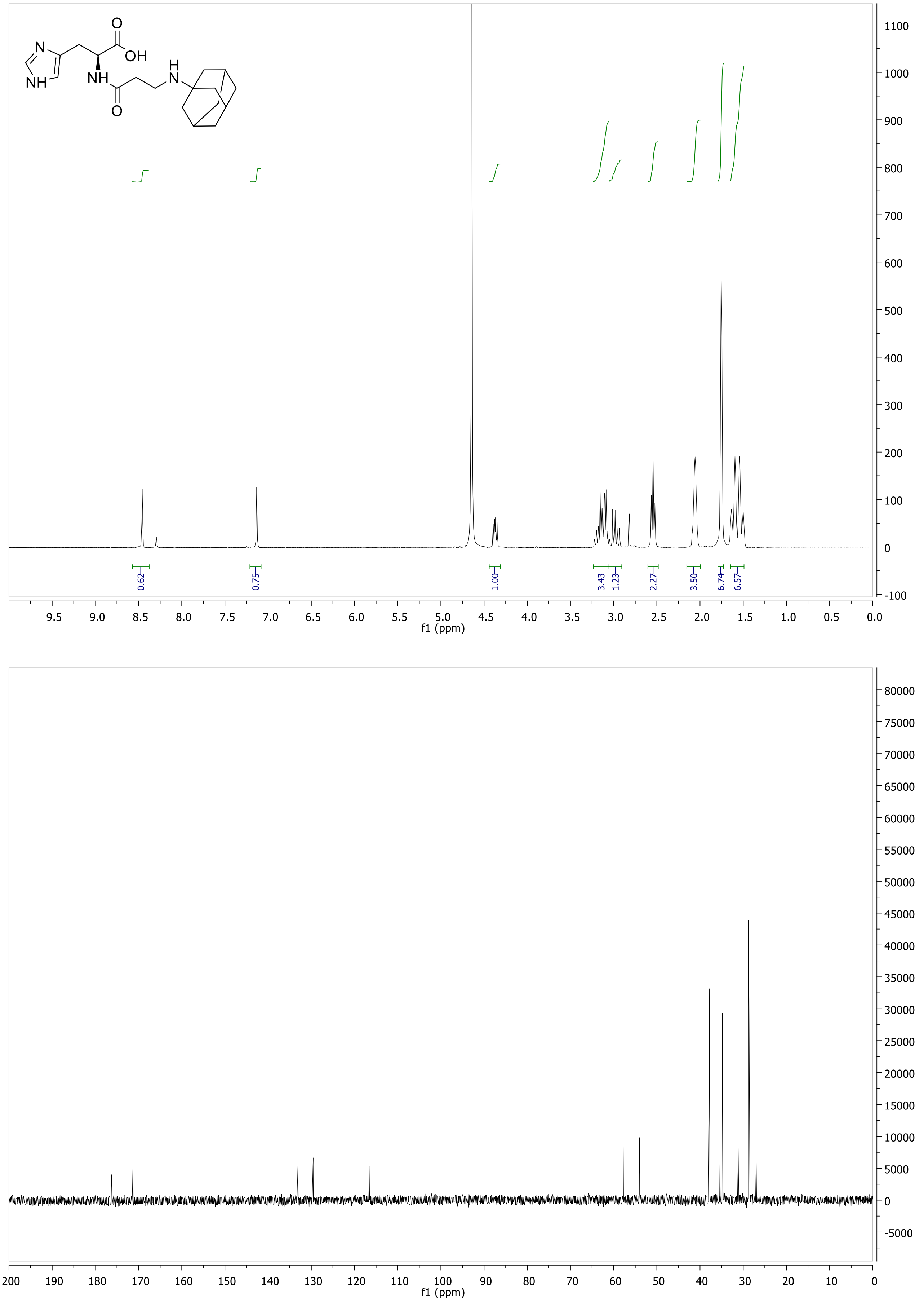

Figure S5. ^{1}H- and ^{13}C NMR spectra of compound **V** (D_2O)

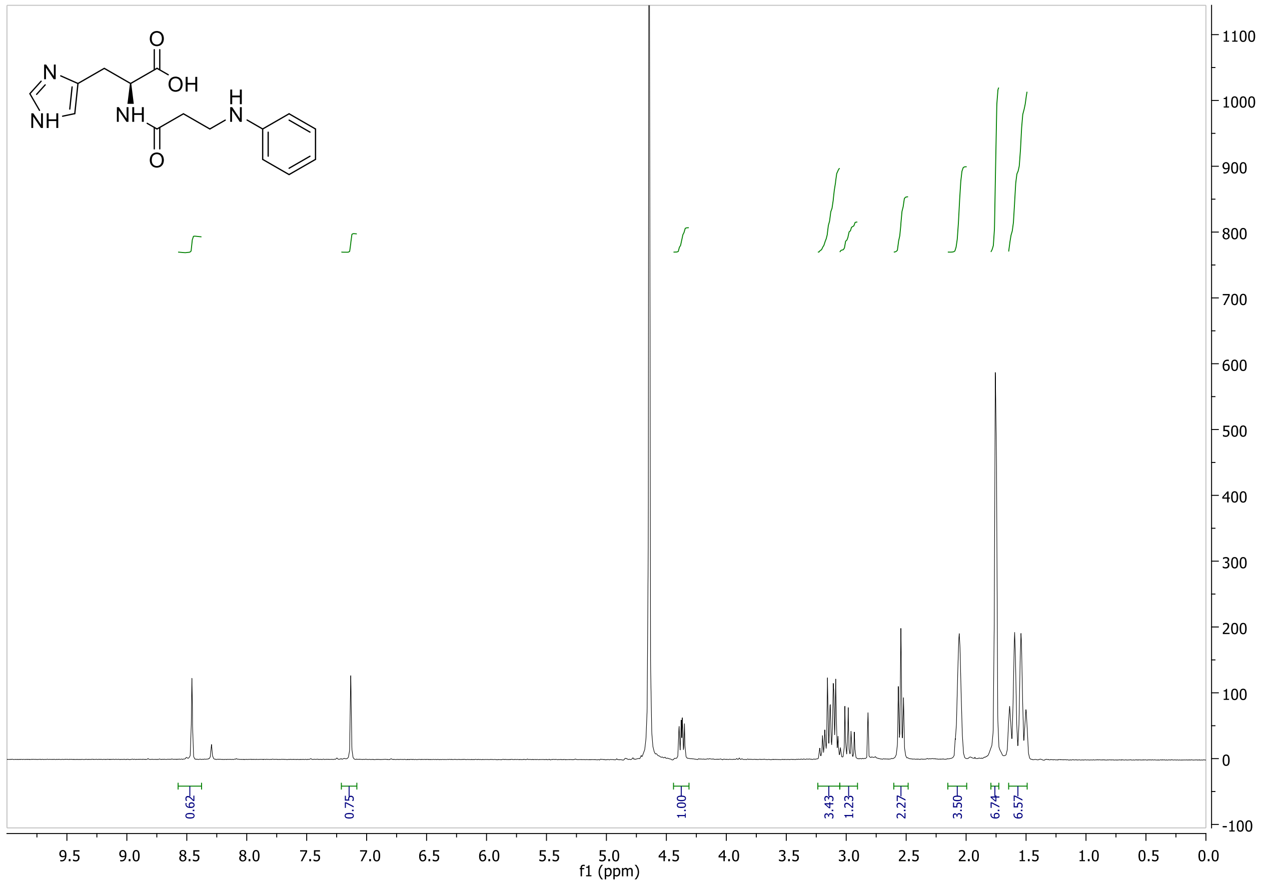

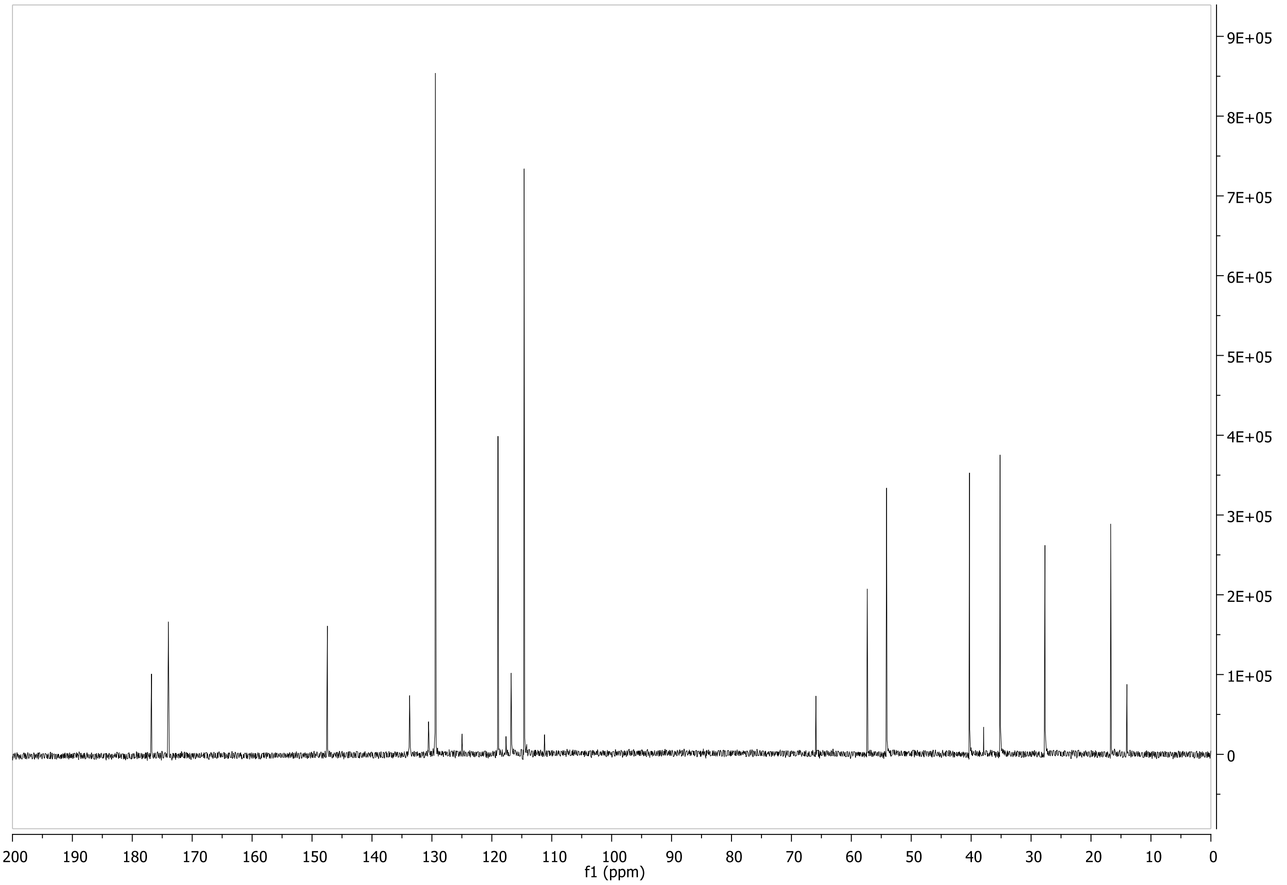

Figure S6. ^{1}H- and ^{13}C NMR spectra of compound **VI** (D_2O)

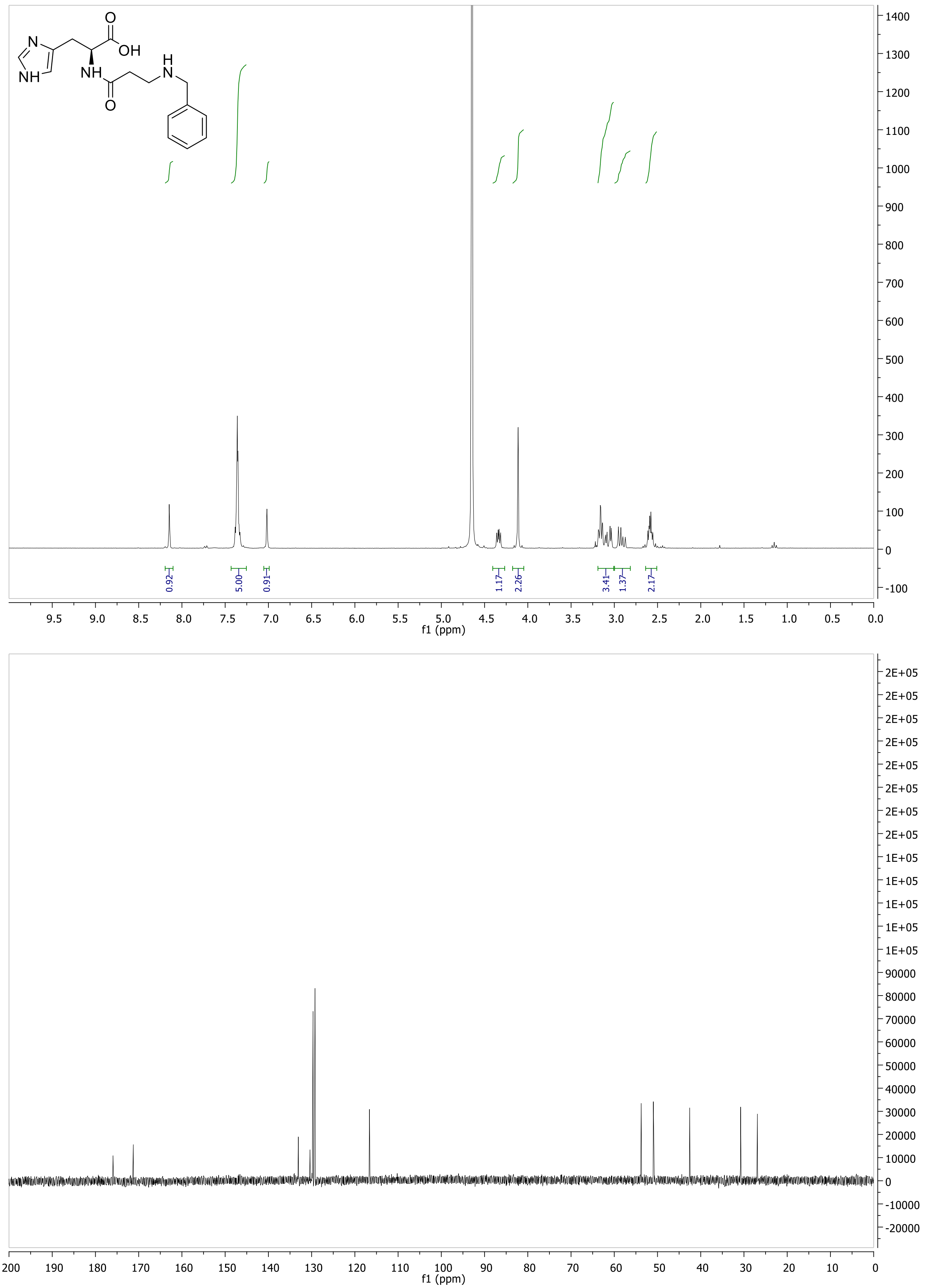

Figure S7. ^{1}H- and ^{13}C NMR spectra of compound **VII** (CD_3OD)

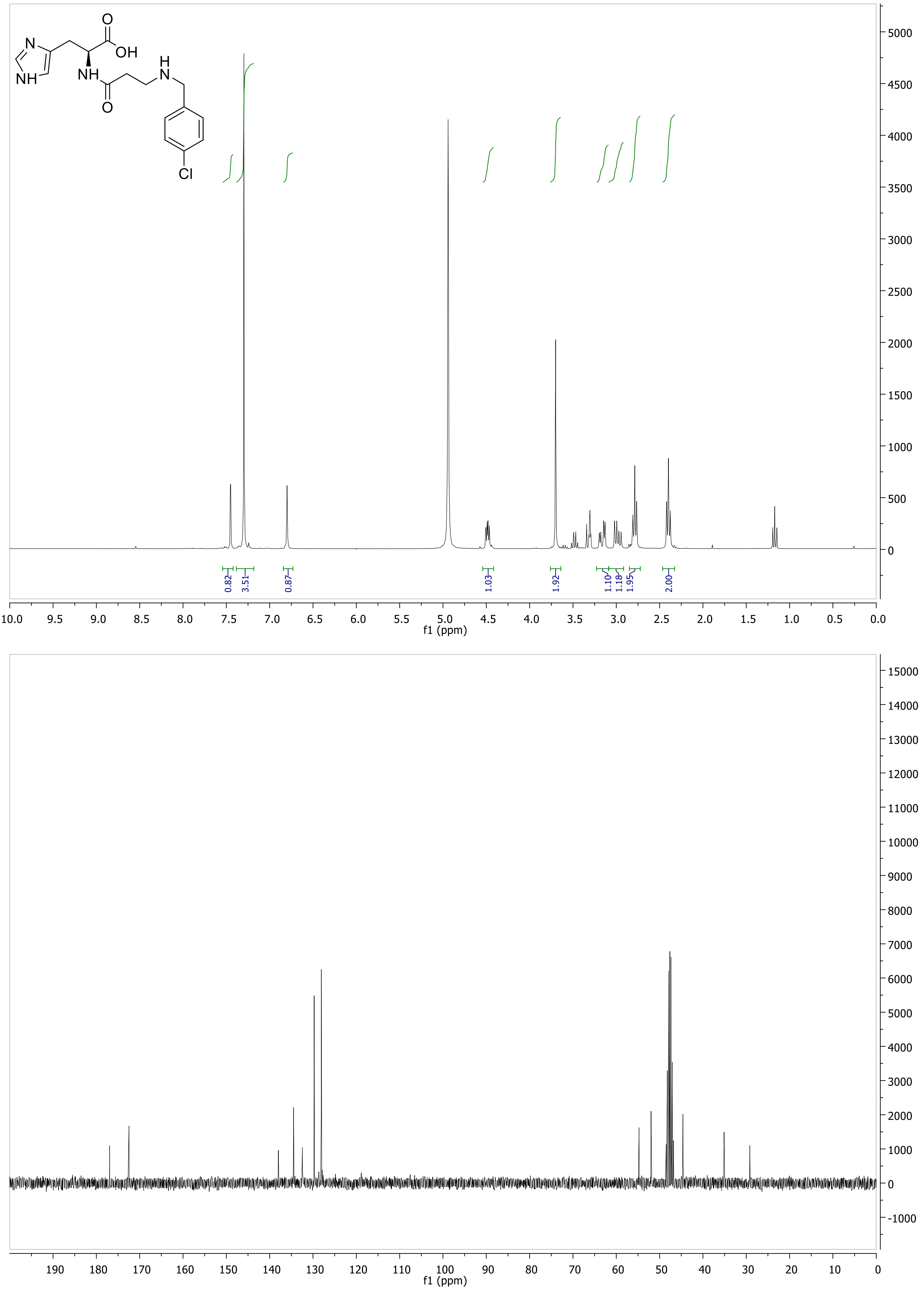

Figure S8. ^{1}H- and ^{13}C NMR spectra of compound **VIII** (CD_3OD)

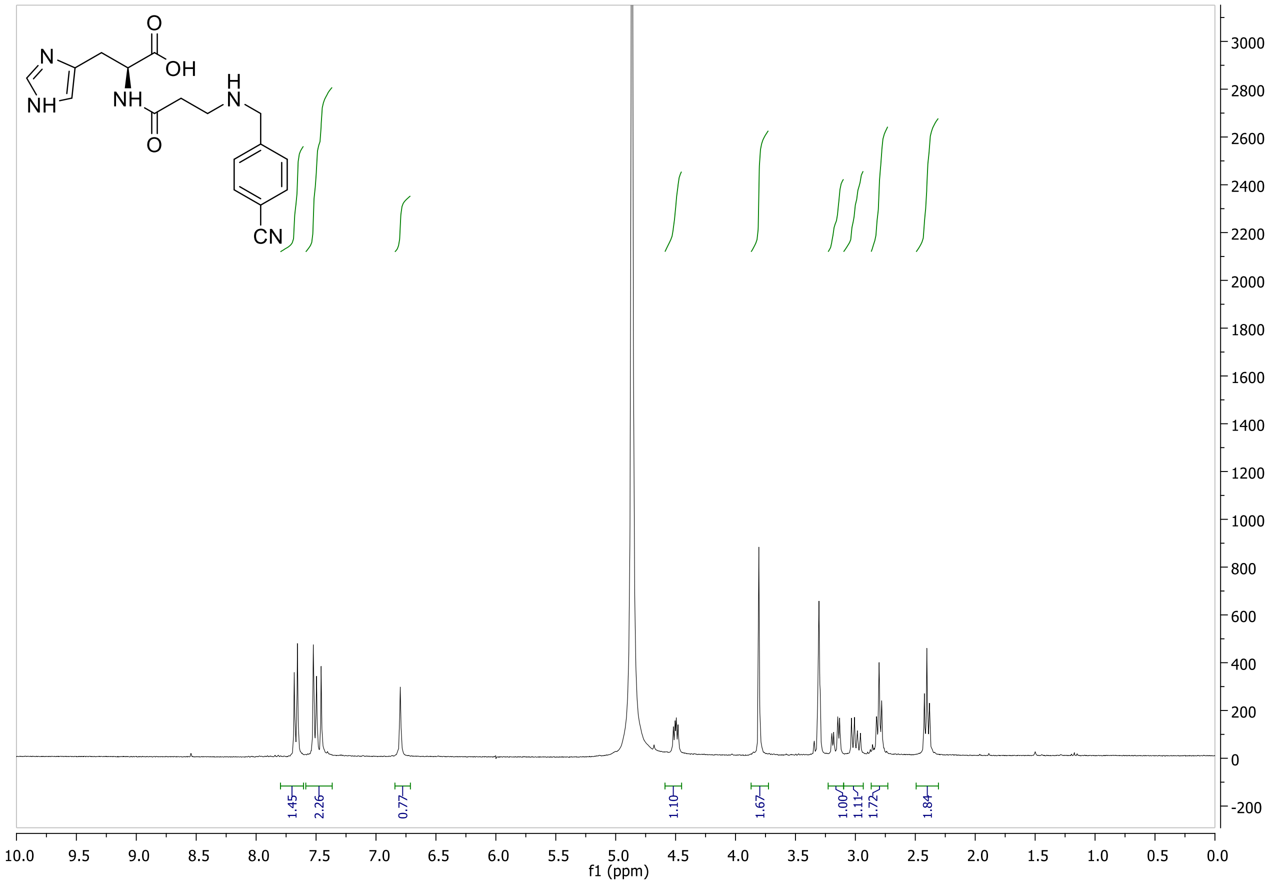

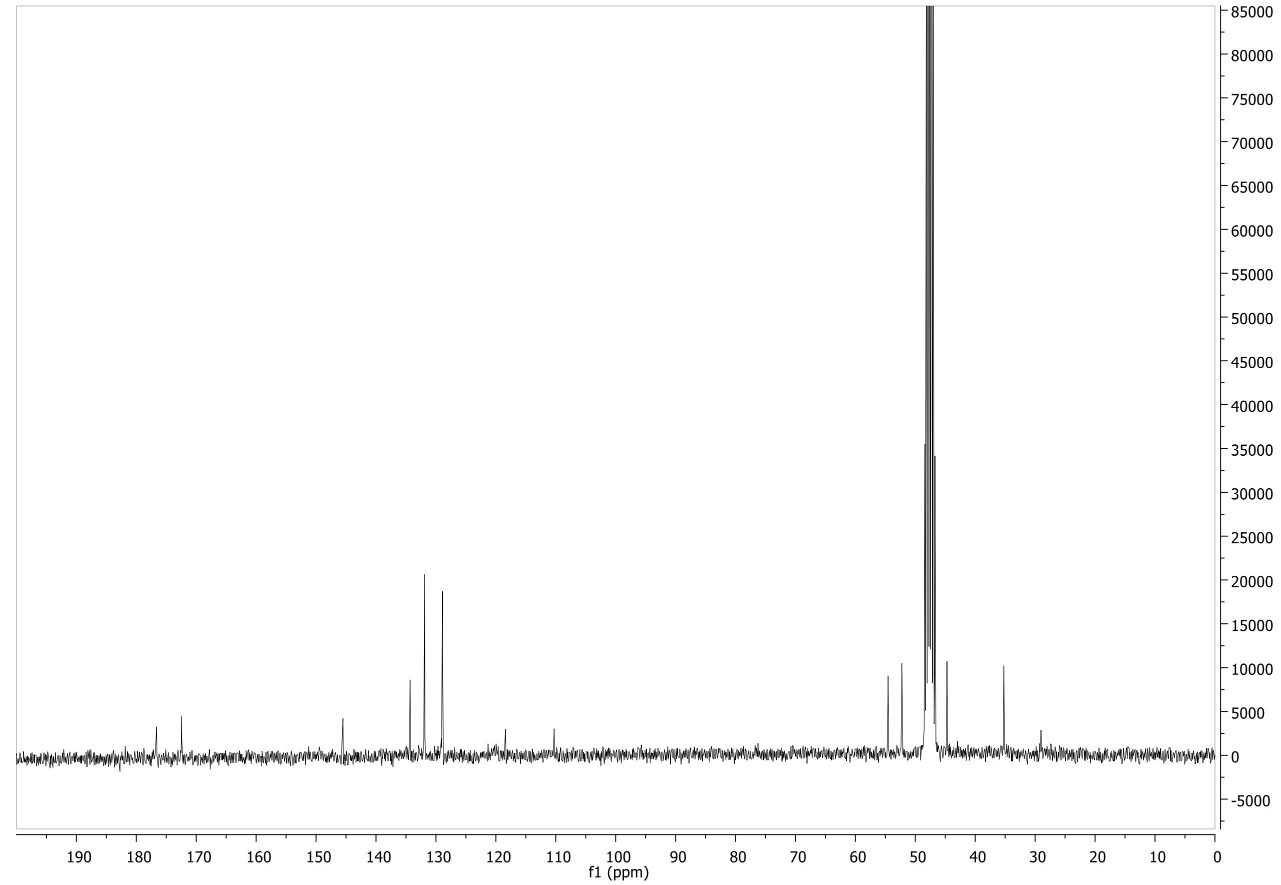

Figure S9. ^{1}H- and ^{13}C NMR spectra of compound **IX** (CD_3OD)

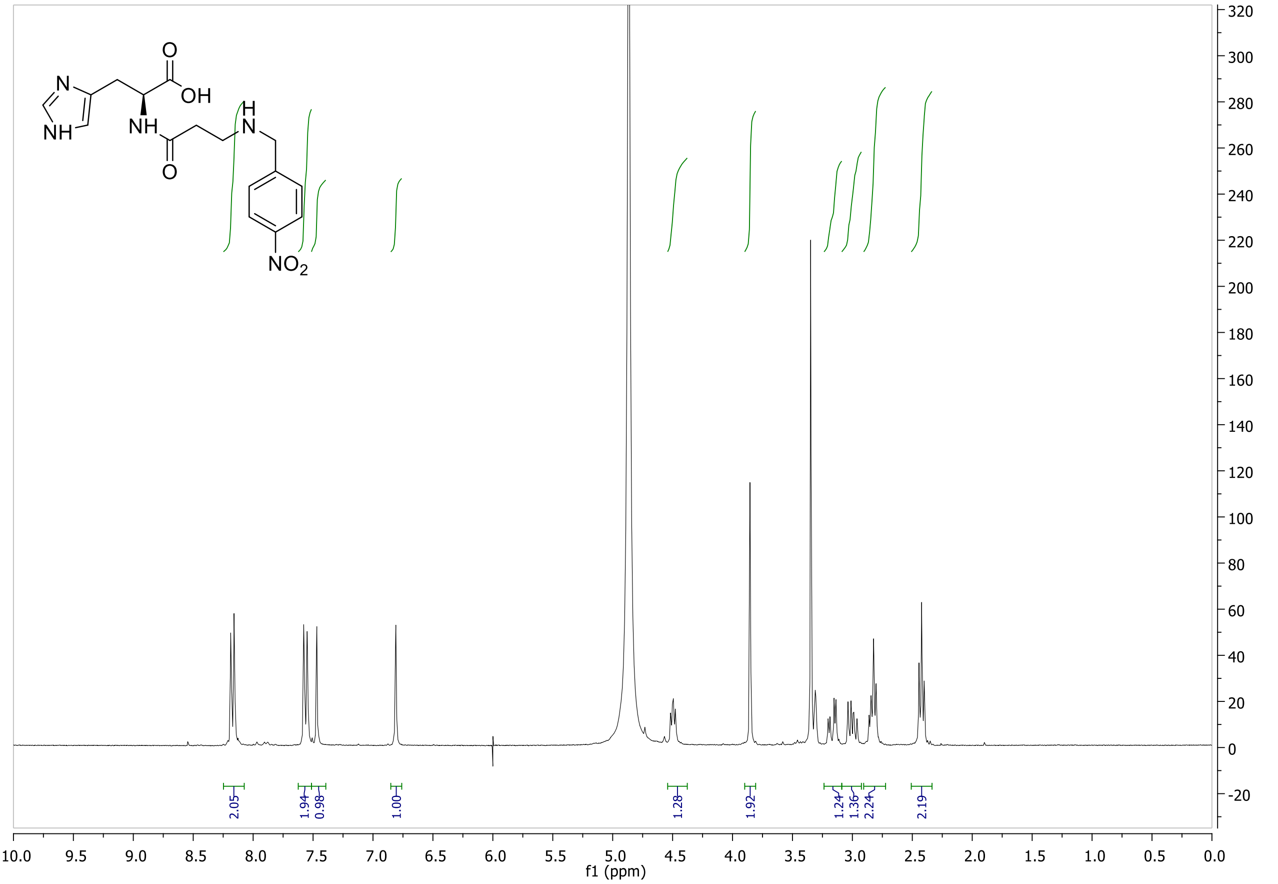

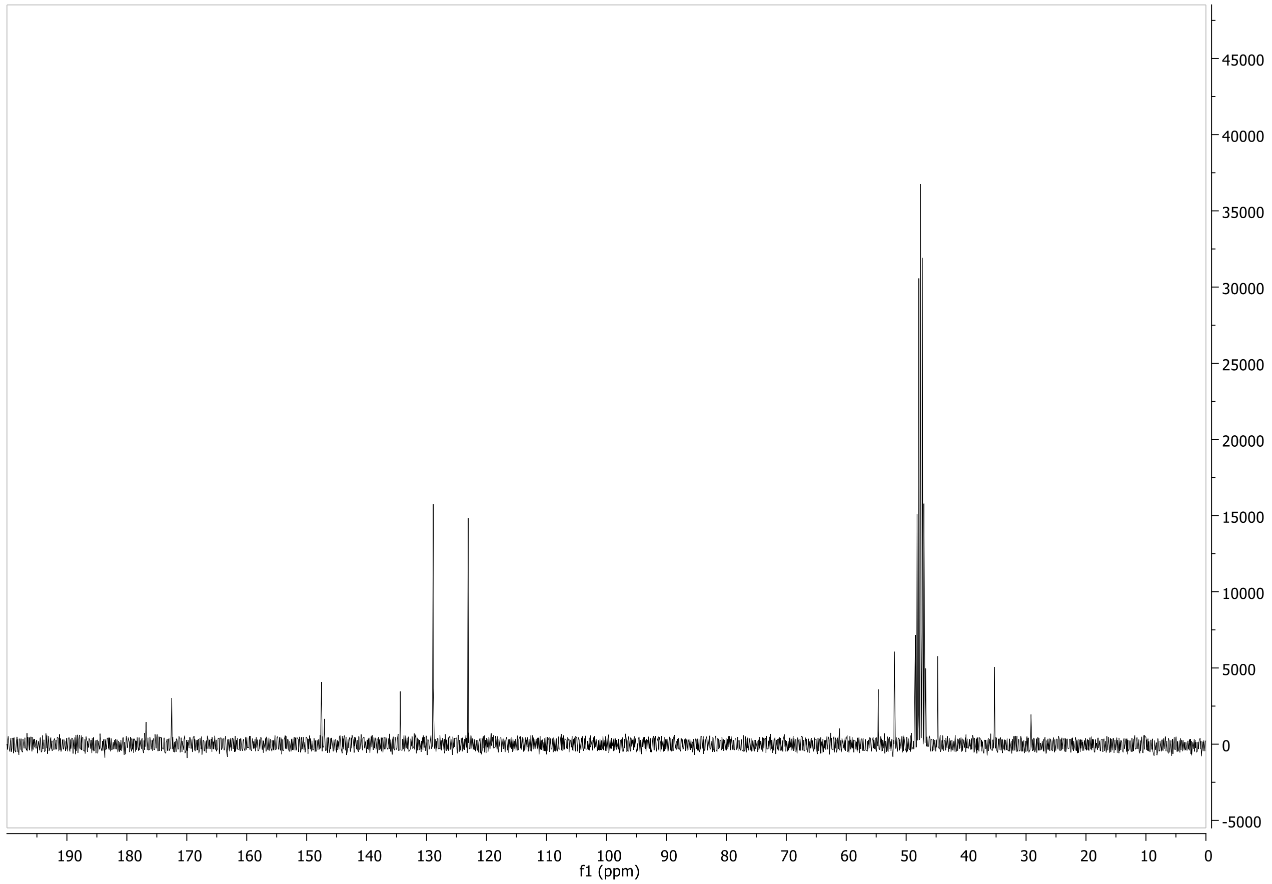

Table S1. Hirshfeld charges, condensed Fukui functions and condensed dual descriptors for **L-CAR**. Units used below are "e" (elementary charge)

Atom	q(N)	q(N+1)	q(N-1)	f_k-	f_k+	f_k0	CDD
1(N)	-0.0798	-0.1001	-0.0715	0.0083	0.0203	0.0143	0.012
2(H)	0.1242	0.0955	0.1347	0.0105	0.0287	0.0196	0.0182
3(C)	0.0333	0.0128	0.0354	0.0021	0.0204	0.0113	0.0183
4(H)	0.0476	0.0297	0.0516	0.0039	0.0179	0.0109	0.014
5(C)	-0.0496	-0.0628	-0.0337	0.0159	0.0131	0.0145	-0.0028
6(H)	0.0366	0.0125	0.0647	0.0281	0.0242	0.0261	-0.004
7(H)	0.0321	0.0171	0.0557	0.0235	0.015	0.0193	-0.0085
8(C)	0.0098	0.0087	0.1048	0.095	0.0011	0.048	-0.0939
9(N)	-0.0482	-0.0678	-0.0153	0.0329	0.0196	0.0262	-0.0133
10(C)	-0.0448	-0.0579	0.053	0.0977	0.0131	0.0554	-0.0846
11(H)	0.1468	0.1286	0.1762	0.0294	0.0183	0.0238	-0.0111
12(C)	0.0352	-0.0198	0.1398	0.1046	0.055	0.0798	-0.0495
13(N)	-0.2071	-0.2463	-0.1421	0.065	0.0392	0.0521	-0.0258
14(H)	0.0455	0.0373	0.0842	0.0387	0.0082	0.0235	-0.0304
15(H)	0.0558	0.0255	0.1007	0.0449	0.0303	0.0376	-0.0146
16(C)	0.1663	0.1068	0.1799	0.0136	0.0595	0.0365	0.0458
17(O)	-0.2973	-0.3635	-0.2504	0.0469	0.0662	0.0565	0.0193
18(C)	-0.0542	-0.0773	-0.0434	0.0109	0.023	0.0169	0.0122
19(C)	-0.0064	-0.0224	0.0154	0.0218	0.0161	0.0189	-0.0057
20(H)	0.0265	0	0.0379	0.0114	0.0265	0.019	0.0152
21(H)	0.0457	0.0049	0.0618	0.0161	0.0408	0.0285	0.0246
22(H)	0.0158	-0.0084	0.0482	0.0325	0.0241	0.0283	-0.0084
23(H)	0.0375	0.0227	0.0557	0.0182	0.0148	0.0165	-0.0034
**24(N)**	**-0.2131**	**-0.2361**	**-0.0977**	**0.1154**	**0.023**	**0.0692**	**-0.0924**
25(C)	0.2041	0.0965	0.2015	-0.0025	0.1076	0.0525	0.1101
26(O)	-0.2833	-0.3987	-0.266	0.0174	0.1153	0.0664	0.098
27(O)	-0.1567	-0.2173	-0.1477	0.009	0.0605	0.0348	0.0515
28(H)	0.1875	0.1505	0.2016	0.0141	0.037	0.0256	0.0229
29(H)	0.091	0.0609	0.1279	0.0368	0.0302	0.0335	-0.0067
30(H)	0.099	0.0681	0.137	0.038	0.0309	0.0345	-0.0072

Table S2. Hirshfeld charges, condensed Fukui functions and condensed dual descriptors for compound **I**. Units used below are "e" (elementary charge)

Atom	q(N)	q(N+1)	q(N-1)	f_k-	f_k+	f_k0	CDD
1(N)	-0.0792	-0.0982	-0.0734	0.0058	0.019	0.0124	0.0132
2(H)	0.1244	0.1021	0.1324	0.008	0.0223	0.0152	0.0143
3(C)	0.0335	0.0135	0.0356	0.0021	0.02	0.011	0.0179
4(H)	0.0475	0.0306	0.0511	0.0036	0.0169	0.0103	0.0133
5(C)	-0.0496	-0.0643	-0.036	0.0135	0.0147	0.0141	0.0012
6(H)	0.0375	0.0103	0.0612	0.0237	0.0272	0.0255	0.0034
7(H)	0.032	0.0175	0.052	0.02	0.0145	0.0173	-0.0055
8(C)	0.009	0.0085	0.093	0.084	0.0006	0.0423	-0.0834
9(N)	-0.0484	-0.0677	-0.0193	0.029	0.0194	0.0242	-0.0097
10(C)	-0.0446	-0.0576	0.0417	0.0862	0.013	0.0496	-0.0732
11(H)	0.1468	0.1242	0.1728	0.026	0.0226	0.0243	-0.0033
12(C)	0.0352	-0.0177	0.1282	0.093	0.0529	0.073	-0.0401
13(N)	-0.2072	-0.2452	-0.1494	0.0578	0.038	0.0479	-0.0198
14(H)	0.0454	0.0373	0.0797	0.0342	0.0081	0.0212	-0.0261
15(H)	0.0558	0.0261	0.096	0.0402	0.0298	0.035	-0.0104
16(C)	0.1671	0.1052	0.1748	0.0077	0.0619	0.0348	0.0543
17(O)	-0.2974	-0.365	-0.2712	0.0262	0.0676	0.0469	0.0414
18(C)	-0.0534	-0.0748	-0.0459	0.0075	0.0214	0.0144	0.0139
19(C)	-0.0069	-0.0179	0.0145	0.0214	0.0111	0.0162	-0.0103
20(H)	0.027	0.0051	0.0366	0.0096	0.0219	0.0157	0.0123
21(H)	0.0457	0.005	0.0596	0.0138	0.0407	0.0273	0.0269
22(H)	0.0141	-0.0025	0.05	0.0359	0.0166	0.0263	-0.0193
23(H)	0.0364	0.0243	0.0563	0.02	0.012	0.016	-0.008
**24(N)**	**-0.1565**	**-0.1682**	**-0.0224**	**0.134**	**0.0117**	**0.0729**	**-0.1223**
25(C)	0.2048	0.0941	0.2025	-0.0023	0.1108	0.0542	0.1131
26(O)	-0.2842	-0.4012	-0.2682	0.0159	0.117	0.0665	0.1011
27(O)	-0.1562	-0.2193	-0.1485	0.0078	0.063	0.0354	0.0553
28(H)	0.1882	0.1506	0.2007	0.0125	0.0376	0.025	0.0251
29(H)	0.0912	0.0689	0.1322	0.0409	0.0223	0.0316	-0.0186
30(C)	-0.0421	-0.0572	-0.0126	0.0295	0.0151	0.0223	-0.0144
31(H)	0.0338	0.0114	0.0599	0.0262	0.0223	0.0242	-0.0038
32(H)	0.016	0.0007	0.0569	0.0409	0.0153	0.0281	-0.0256
33(H)	0.0343	0.0216	0.0597	0.0254	0.0127	0.0191	-0.0127

Table S3. Hirshfeld charges, condensed Fukui functions and condensed dual descriptors for compound **VI**. Units used below are "e" (elementary charge)

Atom	q(N)	q(N+1)	q(N-1)	f_k-	f_k+	f_k0	CDD
1(N)	-0.0792	-0.0905	-0.0711	0.0081	0.0113	0.0097	0.0032
2(H)	0.124	0.1117	0.1317	0.0077	0.0123	0.01	0.0046
3(C)	0.0334	0.0196	0.0359	0.0025	0.0138	0.0082	0.0113
4(H)	0.048	0.0355	0.0518	0.0037	0.0126	0.0082	0.0088
5(C)	-0.0498	-0.0572	-0.0387	0.0111	0.0074	0.0093	-0.0036
6(H)	0.0369	0.0215	0.0561	0.0193	0.0154	0.0173	-0.0039
7(H)	0.032	0.0239	0.0482	0.0162	0.0081	0.0122	-0.0081
8(C)	0.0099	0.0136	0.0759	0.066	-0.0037	0.0312	-0.0696
9(N)	-0.048	-0.0576	-0.0245	0.0235	0.0096	0.0165	-0.0138
10(C)	-0.0448	-0.051	0.024	0.0688	0.0062	0.0375	-0.0625
11(H)	0.1469	0.1374	0.1681	0.0212	0.0094	0.0153	-0.0117
12(C)	0.0353	0.004	0.111	0.0757	0.0313	0.0535	-0.0444
13(N)	-0.207	-0.2297	-0.16	0.047	0.0227	0.0348	-0.0244
14(H)	0.0456	0.0418	0.0729	0.0273	0.0037	0.0155	-0.0236
15(H)	0.0559	0.0379	0.0891	0.0332	0.018	0.0256	-0.0152
16(C)	0.165	0.1344	0.1759	0.0109	0.0305	0.0207	0.0196
17(O)	-0.3001	-0.3391	-0.2663	0.0337	0.039	0.0364	0.0053
18(C)	-0.0608	-0.0682	-0.0452	0.0156	0.0073	0.0115	-0.0083
19(C)	-0.0035	-0.0128	0.0114	0.0149	0.0093	0.0121	-0.0056
20(H)	0.0407	0.0306	0.0511	0.0104	0.0101	0.0102	-0.0004
21(H)	0.0246	0.0161	0.0355	0.0108	0.0086	0.0097	-0.0023
22(H)	0.0378	0.0288	0.0523	0.0145	0.009	0.0118	-0.0056
23(H)	0.0312	0.0215	0.0477	0.0164	0.0098	0.0131	-0.0067
**24(N)**	**-0.1505**	**-0.1617**	**-0.0493**	**0.1012**	**0.0112**	**0.0562**	**-0.0901**
25(C)	0.204	0.1244	0.2021	-0.0018	0.0796	0.0389	0.0814
26(O)	-0.2825	-0.3655	-0.2691	0.0134	0.083	0.0482	0.0696
27(O)	-0.1578	-0.2008	-0.1515	0.0062	0.0431	0.0246	0.0369
28(H)	0.1875	0.1622	0.1981	0.0106	0.0253	0.018	0.0148
29(C)	-0.0086	-0.0212	0.0074	0.0161	0.0126	0.0143	-0.0035
30(H)	0.0356	0.0187	0.0541	0.0184	0.0169	0.0177	-0.0016
31(C)	0.0001	-0.0489	0.0046	0.0045	0.0489	0.0267	0.0444
32(C)	-0.0371	-0.0818	-0.0086	0.0285	0.0446	0.0366	0.0161
33(C)	-0.047	-0.0752	-0.0341	0.0129	0.0282	0.0205	0.0152
34(C)	-0.0392	-0.0731	-0.0149	0.0243	0.0339	0.0291	0.0096
35(H)	0.0421	0.0171	0.0544	0.0122	0.0251	0.0187	0.0129
36(C)	-0.0427	-0.0911	-0.0029	0.0398	0.0483	0.0441	0.0085
37(H)	0.0345	0.0151	0.0445	0.01	0.0194	0.0147	0.0094
38(C)	-0.0415	-0.111	-0.0098	0.0317	0.0695	0.0506	0.0377
39(H)	0.0414	0.0172	0.0592	0.0178	0.0242	0.021	0.0065
40(H)	0.0399	0.0116	0.0598	0.0199	0.0283	0.0241	0.0084
41(H)	0.0408	0.0055	0.0603	0.0195	0.0353	0.0274	0.0158
42(H)	0.0169	0.0057	0.0428	0.0259	0.0112	0.0185	-0.0147

43(H)	0.0902	0.0805	0.1204	0.0303	0.0096	0.0199	-0.0207

Table S4. Hirshfeld charges, condensed Fukui functions and condensed dual descriptors for compound **VII**. Units used below are "e" (elementary charge)

Atom	q(N)	q(N+1)	q(N-1)	f_k-	f_k+	f_k0	CDD
1(N)	-0.0789	-0.0892	-0.0715	0.0074	0.0103	0.0088	0.0029
2(H)	0.1242	0.1134	0.1316	0.0073	0.0109	0.0091	0.0035
3(C)	0.0337	0.0219	0.0358	0.0021	0.0118	0.0069	0.0097
4(H)	0.0482	0.0371	0.0517	0.0035	0.0111	0.0073	0.0075
5(C)	-0.0497	-0.0559	-0.0386	0.0112	0.0062	0.0087	-0.0049
6(H)	0.0366	0.0242	0.056	0.0195	0.0123	0.0159	-0.0071
7(H)	0.0322	0.0254	0.0487	0.0165	0.0067	0.0116	-0.0098
8(C)	0.0098	0.0144	0.0766	0.0668	-0.0045	0.0311	-0.0713
9(N)	-0.0481	-0.0555	-0.0247	0.0235	0.0074	0.0154	-0.016
10(C)	-0.0448	-0.0492	0.0243	0.0692	0.0044	0.0368	-0.0648
11(H)	0.1469	0.1392	0.1681	0.0212	0.0077	0.0145	-0.0135
12(C)	0.0358	0.0103	0.1116	0.0758	0.0255	0.0506	-0.0503
13(N)	-0.2067	-0.2253	-0.1597	0.047	0.0186	0.0328	-0.0284
14(H)	0.0453	0.0428	0.0727	0.0274	0.0024	0.0149	-0.025
15(H)	0.0561	0.0411	0.0892	0.0331	0.0151	0.0241	-0.018
16(C)	0.1647	0.1387	0.1755	0.0108	0.026	0.0184	0.0152
17(O)	-0.3004	-0.334	-0.2667	0.0336	0.0336	0.0336	0
18(C)	-0.0607	-0.0673	-0.0474	0.0134	0.0066	0.01	-0.0068
19(C)	-0.0028	-0.01	0.0099	0.0127	0.0072	0.01	-0.0055
20(H)	0.0409	0.0315	0.0501	0.0093	0.0094	0.0093	0.0001
21(H)	0.0247	0.0175	0.0344	0.0096	0.0073	0.0084	-0.0023
22(H)	0.0386	0.0307	0.0508	0.0123	0.0079	0.0101	-0.0044
23(H)	0.0316	0.0232	0.0453	0.0137	0.0084	0.0111	-0.0053
**24(N)**	**-0.1498**	**-0.1571**	**-0.0718**	**0.078**	**0.0073**	**0.0426**	**-0.0707**
25(C)	0.2039	0.1347	0.202	-0.0019	0.0692	0.0336	0.0712
26(O)	-0.2818	-0.3542	-0.269	0.0128	0.0725	0.0426	0.0597
27(O)	-0.1574	-0.1947	-0.1513	0.0061	0.0373	0.0217	0.0311
28(H)	0.1877	0.1659	0.198	0.0103	0.0218	0.016	0.0115
29(C)	-0.0075	-0.0179	0.0066	0.0141	0.0104	0.0123	-0.0037
30(H)	0.0372	0.0202	0.0555	0.0182	0.017	0.0176	-0.0013
31(C)	0.0009	-0.0345	0.0199	0.019	0.0354	0.0272	0.0163
32(C)	-0.0287	-0.103	-0.0093	0.0195	0.0742	0.0468	0.0548
33(C)	-0.0388	-0.0722	-0.0253	0.0135	0.0334	0.0234	0.0199
34(C)	-0.0425	-0.0801	-0.0236	0.0189	0.0376	0.0282	0.0187
35(H)	0.0479	0.0132	0.0592	0.0114	0.0346	0.023	0.0233
36(C)	-0.0461	-0.1227	-0.0147	0.0314	0.0766	0.054	0.0452
37(H)	0.0405	0.0179	0.0512	0.0108	0.0226	0.0167	0.0118
38(C)	0.0237	-0.0172	0.0538	0.0301	0.0409	0.0355	0.0108
39(H)	0.0484	0.0233	0.0639	0.0154	0.0251	0.0203	0.0097
40(H)	0.0471	0.0101	0.0642	0.0171	0.037	0.0271	0.0198
41(H)	0.0187	0.007	0.0396	0.0209	0.0118	0.0163	-0.0091
42(H)	0.0909	0.0815	0.1154	0.0244	0.0094	0.0169	-0.015

43(Cl)	-0.0711	-0.1449	0.0122	0.0833	0.0738	0.0786	-0.0096

Table S5. Hirshfeld charges, condensed Fukui functions and condensed dual descriptors for compound **VIII**. Units used below are "e" (elementary charge)

Atom	q(N)	q(N+1)	q(N-1)	f_k-	f_k+	f_k0	CDD
1(N)	-0.0786	-0.0857	-0.0704	0.0082	0.0071	0.0076	-0.0011
2(H)	0.1243	0.1184	0.1326	0.0083	0.0059	0.0071	-0.0023
3(C)	0.0338	0.026	0.036	0.0023	0.0077	0.005	0.0055
4(H)	0.0483	0.0407	0.052	0.0037	0.0076	0.0057	0.0039
5(C)	-0.0497	-0.0531	-0.0374	0.0123	0.0034	0.0079	-0.0089
6(H)	0.0366	0.0302	0.0581	0.0215	0.0063	0.0139	-0.0152
7(H)	0.0322	0.0285	0.0504	0.0182	0.0038	0.011	-0.0144
8(C)	0.0097	0.0145	0.0832	0.0735	-0.0049	0.0343	-0.0784
9(N)	-0.048	-0.0517	-0.0222	0.0258	0.0037	0.0147	-0.0221
10(C)	-0.0447	-0.0469	0.0311	0.0759	0.0021	0.039	-0.0737
11(H)	0.147	0.1427	0.1702	0.0232	0.0043	0.0137	-0.0189
12(C)	0.0361	0.0207	0.1188	0.0827	0.0154	0.049	-0.0673
13(N)	-0.2064	-0.218	-0.1551	0.0513	0.0116	0.0315	-0.0397
14(H)	0.0453	0.0445	0.0754	0.0301	0.0008	0.0155	-0.0293
15(H)	0.0564	0.0466	0.0923	0.0359	0.0097	0.0228	-0.0262
16(C)	0.1648	0.1516	0.1771	0.0123	0.0132	0.0127	0.0008
17(O)	-0.2998	-0.3211	-0.2616	0.0382	0.0214	0.0298	-0.0168
18(C)	-0.0606	-0.0649	-0.0451	0.0155	0.0043	0.0099	-0.0111
19(C)	-0.0021	-0.0108	0.0116	0.0137	0.0087	0.0112	-0.0049
20(H)	0.0411	0.0367	0.0519	0.0109	0.0044	0.0076	-0.0065
21(H)	0.0251	0.0197	0.0359	0.0108	0.0053	0.0081	-0.0055
22(H)	0.0391	0.0308	0.0526	0.0135	0.0083	0.0109	-0.0052
23(H)	0.0329	0.0213	0.0472	0.0143	0.0115	0.0129	-0.0028
**24(N)**	**-0.1482**	**-0.1566**	**-0.0561**	**0.0921**	**0.0084**	**0.0503**	**-0.0838**
25(C)	0.2039	0.1608	0.2018	-0.0022	0.0431	0.0205	0.0453
26(O)	-0.2815	-0.3279	-0.2677	0.0138	0.0465	0.0301	0.0327
27(O)	-0.1574	-0.1803	-0.1507	0.0068	0.0229	0.0148	0.0162
28(H)	0.1879	0.1738	0.199	0.0111	0.0141	0.0126	0.003
29(C)	-0.0063	-0.0215	0.0083	0.0146	0.0152	0.0149	0.0006
30(H)	0.0396	0.0186	0.0563	0.0167	0.021	0.0189	0.0043
31(C)	0.0139	-0.0592	0.0187	0.0048	0.0731	0.0389	0.0682
32(C)	-0.0273	-0.0738	-0.0129	0.0144	0.0465	0.0304	0.0321
33(C)	-0.0373	-0.0799	-0.0289	0.0085	0.0426	0.0255	0.0341
34(C)	-0.019	-0.0707	-0.0046	0.0144	0.0516	0.033	0.0373
35(H)	0.0499	0.02	0.0576	0.0076	0.03	0.0188	0.0223
36(C)	-0.0217	-0.0804	0.0026	0.0243	0.0587	0.0415	0.0344
37(H)	0.0428	0.0139	0.05	0.0073	0.0289	0.0181	0.0216
38(C)	0.0101	-0.0569	0.0354	0.0253	0.067	0.0462	0.0417
39(H)	0.0549	0.0238	0.0676	0.0127	0.0311	0.0219	0.0184
40(H)	0.0537	0.0207	0.0677	0.014	0.033	0.0235	0.019
41(H)	0.0204	0.0059	0.0433	0.0229	0.0145	0.0187	-0.0083
42(H)	0.0923	0.0834	0.12	0.0278	0.0089	0.0183	-0.0189

43(C)	0.0627	0.0042	0.0755	0.0128	0.0585	0.0357	0.0457
44(N)	-0.2159	-0.3386	-0.1674	0.0484	0.1227	0.0856	0.0743

Table S6. Hirshfeld charges, condensed Fukui functions and condensed dual descriptors for compound **IX**. Units used below are "e" (elementary charge)

Atom	q(N)	q(N+1)	q(N-1)	f_k-	f_k+	f_k0	CDD
1(N)	-0.0782	-0.0828	-0.0692	0.009	0.0046	0.0068	-0.0044
2(H)	0.1244	0.1216	0.1334	0.0089	0.0028	0.0059	-0.0061
3(C)	0.0336	0.0291	0.0364	0.0028	0.0044	0.0036	0.0016
4(H)	0.0477	0.0432	0.0517	0.004	0.0045	0.0042	0.0005
5(C)	-0.0493	-0.0517	-0.0367	0.0126	0.0023	0.0075	-0.0103
6(H)	0.0379	0.0347	0.0602	0.0222	0.0032	0.0127	-0.019
7(H)	0.0321	0.0299	0.0506	0.0185	0.0022	0.0104	-0.0162
8(C)	0.0082	0.012	0.0846	0.0764	-0.0038	0.0363	-0.0803
9(N)	-0.0482	-0.0504	-0.0214	0.0268	0.0022	0.0145	-0.0247
10(C)	-0.044	-0.0457	0.0345	0.0785	0.0017	0.0401	-0.0768
11(H)	0.1471	0.1445	0.1711	0.024	0.0026	0.0133	-0.0214
12(C)	0.0361	0.0256	0.1218	0.0857	0.0105	0.0481	-0.0752
13(N)	-0.2064	-0.2146	-0.1533	0.0532	0.0081	0.0307	-0.045
14(H)	0.0457	0.0452	0.0768	0.0311	0.0005	0.0158	-0.0306
15(H)	0.0565	0.0496	0.0937	0.0372	0.0069	0.022	-0.0303
16(C)	0.1654	0.1596	0.1784	0.013	0.0058	0.0094	-0.0072
17(O)	-0.3012	-0.314	-0.2614	0.0398	0.0128	0.0263	-0.027
18(C)	-0.0603	-0.063	-0.0432	0.0171	0.0027	0.0099	-0.0144
19(C)	-0.0021	-0.0092	0.0128	0.0149	0.0071	0.011	-0.0078
20(H)	0.0406	0.0389	0.0524	0.0117	0.0017	0.0067	-0.0101
21(H)	0.0258	0.0224	0.0376	0.0118	0.0034	0.0076	-0.0084
22(H)	0.0388	0.032	0.0536	0.0148	0.0068	0.0108	-0.008
23(H)	0.034	0.023	0.0499	0.0159	0.011	0.0135	-0.0049
**24(N)**	**-0.1474**	**-0.1527**	**-0.0403**	**0.107**	**0.0053**	**0.0562**	**-0.1018**
25(C)	0.2052	0.1809	0.2029	-0.0024	0.0243	0.011	0.0267
26(O)	-0.2839	-0.3105	-0.2682	0.0156	0.0266	0.0211	0.011
27(O)	-0.1556	-0.1698	-0.149	0.0066	0.0142	0.0104	0.0076
28(H)	0.189	0.1798	0.2006	0.0116	0.0092	0.0104	-0.0025
29(C)	-0.0059	-0.0188	0.0097	0.0156	0.0129	0.0142	-0.0027
30(H)	0.0406	0.0214	0.0561	0.0155	0.0191	0.0173	0.0036
31(C)	0.0177	-0.0496	0.0114	-0.0063	0.0673	0.0305	0.0736
32(C)	-0.0258	-0.0673	-0.014	0.0119	0.0415	0.0267	0.0296
33(C)	-0.0359	-0.0756	-0.0293	0.0066	0.0397	0.0232	0.0332
34(C)	-0.0258	-0.072	-0.0103	0.0155	0.0461	0.0308	0.0307
35(H)	0.0511	0.0216	0.0565	0.0054	0.0295	0.0174	0.024
36(C)	-0.028	-0.0795	-0.0076	0.0204	0.0515	0.036	0.0311
37(H)	0.044	0.0146	0.0493	0.0053	0.0294	0.0173	0.024
38(C)	0.0232	-0.0113	0.0405	0.0173	0.0345	0.0259	0.0172
39(H)	0.0535	0.026	0.0646	0.0111	0.0274	0.0193	0.0163
40(H)	0.0525	0.0236	0.064	0.0115	0.0289	0.0202	0.0174
41(H)	0.0208	0.0072	0.0465	0.0256	0.0137	0.0196	-0.0119
42(H)	0.0931	0.0861	0.1245	0.0314	0.007	0.0192	-0.0244

43(N)	0.2486	0.1609	0.2526	0.004	0.0877	0.0459	0.0837
44(O)	-0.2066	-0.3471	-0.1866	0.0201	0.1405	0.0803	0.1204
45(O)	-0.2084	-0.3482	-0.1879	0.0205	0.1398	0.0801	0.1193
